# Antibody-Functionalized Halloysite Nanotubes for Targeting Bacterial Cells

**DOI:** 10.1021/acsabm.0c01332

**Published:** 2021-04-11

**Authors:** Ofer Prinz Setter, Ariel Movsowitz, Sarah Goldberg, Ester Segal

**Affiliations:** Department of Biotechnology and Food Engineering, Technion—Israel Institute of Technology, Technion City, Haifa 3200003, Israel

**Keywords:** Halloysite nanotubes, bacteria, hybrid, targeting, E. coli, antibody, flow cytometry

## Abstract

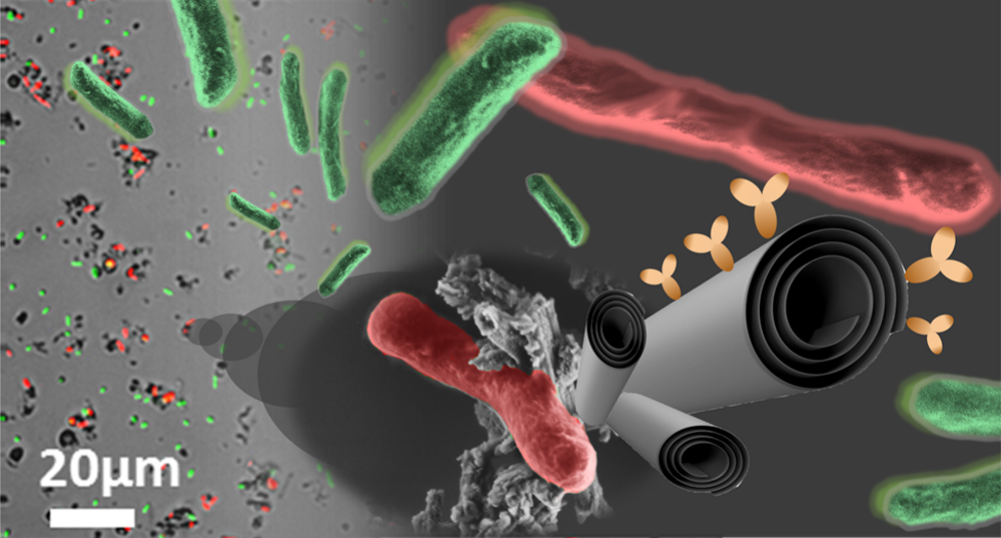

Halloysite nanotubes (HNTs) are naturally occurring tubular clay particles which have emerged in recent years as a promising nanomaterial for numerous applications. Specifically, HNTs’ large pore volume and high specific surface area in combination with their biocompatibility make them ideal nanocarriers for bioactive compounds. This research aims to design and synthesize functionalized HNTs, which could selectively bind to target bacterial cells in suspension. Such a system can allow us to treat target cells within a challenging heterogeneous population, such as contaminated ecosystems or gut flora. HNTs functionalization is achieved by immobilizing specific antibodies onto the nanotube surface. The synthetic route is realized by the following subsequent steps: acidic etching of the HNTs, silanization of reactive surface hydroxyls, conjugation of protein A, and oriented immobilization of the antibody. HNT functionalization is studied by a set of analytical techniques including attenuated total reflectance Fourier-transform infrared spectroscopy, zeta potential measurements, thermal gravimetric analysis, scanning and transmission electron microscopy, as well as fluorescence microscopy. The selective binding of the functionalized HNTs to their target bacteria is observed upon incubation with live homogenous and heterogeneous cultures using fluorescence microscopy and high-throughput flow cytometry. Plate count and live/dead staining experiments demonstrate the biocompatibility of the antibody-HNT hybrid with its target bacteria. The suggested HNT-based smart carrier constitutes a generic platform for targeted delivery that could be selectively tailored against any microorganism by facile antibody adjustment.

## 1 Introduction

In the mid of the persistent pursuit for novel nanomaterials with biomedical applications, Halloysite nanotubes (HNTs) have gained much interest in recent years.^1^ These natural and abundant clay mineral particles comprise an alumina and silica double layer geologically rolled into 600–900 nm long tubes with outer and inner diameters of roughly 50 and 15 nm, respectively.^2,3^ HNTs’ substantial specific area (50–60 m^2^ g^–1^)^4^ and biocompatibility^5−9^ have been extensively exploited for numerous biological applications^10−13^ including antimicrobial activity,^14,15^ drug delivery^2,16−18^ tissue engineering,^12,19^ and imaging.^20^ Targeted delivery was demonstrated in suspension against tumor cells using folate,^21,22^ biotin,^23^ or enzymatically degradable dextrin capping.^24,25^ The immobilization of more specific capture probes, such as antibodies, onto HNTs was exclusively practiced onto surfaces decorated with HNTs for the capture of circulating tumor cells and targeted delivery of anticancer drugs.^26,27^

Previous research concerning interactions of HNTs with microorganisms has solely relied on non-specific adsorption or whole cell encapsulation without any attempt to endow HNTs with targeting capabilities.^7,28,29^ Earliest work by Barr M. dates back to 1957 demonstrating limited bacterial adsorption onto HNTs (2 mg mL^–1^) during 30 min of incubation at pH 6.8.^30^ Later on, HNTs were shown to promote microalgae flocculation^31^ and spontaneously cover yeast cells in water after modification with magnetite.^32^ The unique ability of HNTs (pristine and hydrophobized) to form oil–water Pickering emulsions was also harnessed for marine bioremediation by physically attracting and stimulating the viability of hydrocarbonoclastic bacteria.^33,34^

Hence, we aim to investigate the unexplored utilization of antibody-functionalized HNTs for specific targeting of live bacterial cells. In the research presented herein, superior targeting of bacteria is realized by modifying HNTs with antibodies properly oriented onto covalently bound protein A (PA).^35^ To enhance HNT surface reactivity, acidic etching is performed to enlarge nanotube specific surface area and expose additional reactive hydroxyl groups.^36−38^ The latter is amino-silanized^39^ and subsequently carboxylated to yield a hydrophilic surface,^40^ which readily forms amide bonds with PA amine groups via carbodiimide coupling using 1-ethyl-3-(3-dimethylaminopropyl)carbodiimide/*N*-hydroxysulfosuccinimide (EDC/sulfo-NHS)^27^ (see more details in the Supporting Information). Finally, oriented immobilization of the antibody Fc-region to the PA-modified surface ensures proper antibody configuration, which is crucial for its binding efficacy.^35,41−43^

Successful modification and functionalization of the HNTs are investigated by attenuated total reflectance Fourier-transform infrared (ATR-FTIR) spectroscopy, zeta potential measurements, thermal gravimetric analysis (TGA), and fluorescence immuno-labeling. The targeting capacity of the antibody-functionalized HNTs is studied by fluorescence microscopy and high-throughput flow cytometry analysis. The latter provides a quantitative tool for investigating the binding between the bacteria and the clay particles based on advanced image analysis of up to 3000 micrographs of real-time flowing objects per sample. Moreover, plate count and live/dead cell staining analysis are employed to study the biocompatibility of the developed hybrid.

The proposed multifunctional system has various potential applications from environmental bioremediation to gastroenteritis treatment, where the manipulation of a specific bacterial strain is required among a heterogeneous population. In addition, localized and specific antibacterial activity is beneficial in the struggle against multi-drug-resistant strains.^44,45^ We believe that this work will open the door to novel HNT-based smart nanocarriers that are inexpensive, biocompatible, ecofriendly, and potentially adjustable against any microorganism of choice for selective manipulation.

## 2 Materials and Methods

### 2.1 Chemicals and Materials

HNTs were supplied by NaturalNano (USA) and dried at 150 °C for 3 h prior to use. Concentrated sulfuric acid, aminopropyltriethoxysilane (APTES), succinic anhydride, 1-ethyl-3-(3-dimethylaminopropyl)carbodiimide (EDC), *N*-hydroxysulfosuccinimide sodium salt (sulfo-NHS), and ethanolamine were obtained from Sigma-Aldrich Chemicals (Israel). Solvents including toluene and dimethylformamide (DMF) were purchased from BioLab (Israel), and ethanol absolute was purchased from Gadot Group (Israel). All buffer solutions were prepared with Milli-Q water (18.2 MΩ cm) and filtered through a 0.22 μm membrane prior to use. Phosphate buffer saline (PBS) pH 7.2 0.1 M contained 50 mM disodium hydrogen phosphate (Spectrum Chemicals, USA), 17 mM sodium dihydrogen phosphate (Merck, Germany), and 68 mM sodium chloride (BioLab). 2-(*N*-morpholino)ethanesulfonic acid (MES) buffer pH 6.0 50 mM contained 27 mM MES and 23 mM MES sodium salt; both were purchased from Sigma-Aldrich Chemicals. Protein A (PA) from *Staphylococcus aureus* was obtained from Sigma-Aldrich Chemicals, and anti *Escherichia coli* antibody from rabbit origin was obtained from Virostat (USA). Fluorescein isothiocyanate (FITC)-tagged anti-rabbit immunoglobulin G (IgG) and FITC-tagged anti-mouse IgG were purchased from Jackson ImmunoResearch Laboratories (USA). Bovine Serum Albumin (BSA) was obtained from MP Biomedicals (USA). *E. coli* (K-12) was generously supplied by Prof. Sima Yaron (Technion) and cultured in Luria broth (LB) medium [10 g L^–1^ Bacto tryptone (BD, USA), 5 g L^–1^ Bacto yeast extract (BD) and 5 g L^–1^ sodium chloride]. Fluorescently labeled [green fluorescent protein (GFP)/Ampicillin resistant (Amp)] *E. coli* (K-12) was also generously supplied by Prof. Sima Yaron and cultured in LB medium with 100 μg mL^–1^ ampicillin (Sigma-Aldrich Chemicals). LB plates for culturing were prepared from LB medium with the addition of 15 g L^–1^ Bacto agar (BD). A LIVE/DEAD BacLight Bacterial Viability Kit for microscopy and quantitative assays was purchased from invitrogen by Thermo Fisher Scientific (USA). For *E. coli* cells expressing a red fluorescent protein (RFP), a plasmid encoding an RFP gene under a strong constitutive promoter was elected from the iGEM 2019 Distribution Kit (Biobrick BBa_J04450, gift of the Technion 2019 iGEM team). Molecular biology grade water was obtained from BioLab and *E. coli* One Shot TOP10 chemically competent cells purchased from Invitrogen (USA, catalog number C404003). Magnesium chloride (MgCl_2_) was obtained from Merck (Germany), and magnesium sulfate (MgSO_4_) was obtained from Alfa Aesar (Germany). d-glucose and chloramphenicol (CM) were purchased from Sigma-Aldrich Chemicals. Fluorescently labeled (GFP/streptomycin-resistant (Str)) *Listeria innocua* (*L. innocua*) was also generously supplied by Prof. Sima Yaron (Technion) and cultured in brain and heart infusion (BH) medium [37 g L^–1^ Bacto Brain Heart Infusion (BD)] with 100 μg mL^–1^ streptomycin sulfate (Sigma-Aldrich). BH plates for culturing were prepared from BH medium with the addition of 15 g L^–1^ Bacto agar and 100 μg mL^–1^ streptomycin sulfate.

### 2.2 Acidic Etching

25 g of HNTs was mixed with 166 mL of deionized water and suspended in a round bottom flask. To ensure lumen contact with the solution, the suspension was evacuated for 20 min several times until no bubbling was observed. Then, 34 mL of concentrated sulfuric acid was added to the suspension, and the mixture was reacted under stirring at 105–110 °C for 16 h. Finally, 400 mL of water was added, and the mixture was filtered and washed again in water. The obtained etched HNTs (E-HNTs) were dried at 120 °C for 2 h.^46^

### 2.3 Silanization and Carboxylation of HNTs

Silanization with APTES was realized according to a well-established procedure by Yuan et al.^39^ E-HNTs were ground to a fine powder using an agate mortar and ultrasonically suspended in dry toluene (24 mg mL^–1^) for 2 h. 25 mL of suspension was mixed with 2 mL of APTES, added dropwise, and refluxed overnight using a calcium sulfate drying tube at 120 °C under stirring. The suspension was centrifuged at a centrifugation force of 3260×*g* for 10 min, and the collected silanized HNTs were washed six times with toluene and once with ethanol absolute. Then, the HNTs were dried overnight in a vacuum oven at 60 °C. Subsequent carboxylation was realized using a published method.^40,47^ Briefly, 100 mg of silanized HNTs was ultrasonically suspended in 10 mL of DMF and reacted with 0.1 M succinic anhydride in DMF for 24 h at room temperature (RT). Particles were washed twice with DMF and once with ethanol absolute. Finally, the obtained particles were dried overnight in a vacuum oven at 50 °C.

### 2.4 Functionalization of HNTs with Antibody

Prior to use, the carboxylated HNTs were washed three times with MES buffer 50 mM pH 6.0 and subsequently ultrasonically suspended in MES buffer to a concentration of 20 mg mL^–1^. The suspension was reacted with 200 mM EDC and 200 mM sulfo-NHS for 30 min at RT under vigorous shaking. The highly hydrophilic^48^ and reactive sulfo-NHS modified E-HNTs were rapidly washed with cold MES buffer and ultrasonically suspended (using a Vibra-Cell ultrasonic probe equipped with a microtip, Sonics & Materials Inc. USA). The suspended HNTs (4 mg mL^–1^) were conjugated with 0.8 mg mL^–1^ PA under shaking (700 rpm) for 2 h at RT and then overnight at 4 °C. The resulting PA-conjugated HNTs (PA_Conj_-HNTs) were washed with MES buffer and subsequently blocked with 100 mM ethanolamine in MES buffer. Next, the particles were washed with PBS buffer (0.1 M pH 7.2) and re-suspended in PBS to a concentration of 5 mg mL^–1^. Antibody-oriented immobilization^35^ was realized by incubating the resulting PA_Conj_-HNTs with an anti-*E. coli* antibody (500 μg mL^–1^) under shaking for 2 h at RT and then overnight at 4 °C. The antibody-functionalized HNTs (Ab-PA_Conj_-HNTs) were washed three times with PBS and immediately tested for activity. A negative control for sulfo-NHS activation was prepared by reacting the neat E-HNTs with EDC/sulfo-NHS under the same conditions. As a control for PA_Conj_-HNTs, PA was adsorbed onto the carboxylated HNTs (PA_Ad_-HNTs) by incubation with the protein under the same conditions. Subsequently, the resulting particles were reacted with the antibody solution as performed for Ab-PA_Conj_-E-HNTs.

### 2.5 Zeta Potential Measurements

Prior to measurements, all particles were thoroughly suspended and diluted to a concentration of 0.1 mg mL^–1^ in double-distilled water or PBS 0.1 M pH 7.2. Measurements were carried out at 25 °C using a Malvern Zetasizer Nano ZSP instrument (UK), and the Zetasizer software was used for data analysis by the Smoluchowski model for particles with dimensions that are considerably larger than their Debye length.

### 2.6 Infrared Spectroscopy

The chemical modification of the HNTs was studied by ATR-FTIR spectroscopy using a Thermo 6700 FT-IR instrument (USA) equipped with a Smart iTR diamond ATR device. For semi quantitative representation, all spectra were normalized to the highest peak around 1000–1030 cm^–1^ attributed to Si–O bonds^38,39^ which should not be affected by the chemical modifications involved in this work.

### 2.7 Thermal Gravimetric Analysis

TGA was carried out using a TGA Q5000 instrument (TA Instruments, USA). The heating was performed at a rate of 20 °C min^–1^ up to 600 °C in dynamic high-resolution mode (sensitivity number: 1, resolution: 4). Results were analyzed by Universal Analysis 200 version 4.5A build 4.5.0.5 software.

### 2.8 Fluorescence Immuno-Labeling

Particle suspensions were washed with PBS three times and blocked with BSA (600 μg mL^–1^) for 60 min at RT to minimize non-specific adsorption. Then, particles were incubated with 15 μg mL^–1^ fluorescently tagged anti-rabbit or anti-mouse IgG for 50 min and washed three times with PBS. Subsequently, the samples were observed under a fluorescence microscope (ZEISS Axio Scope A1, Germany) equipped with a ZEISS (Germany) Axiocom MRc camera. A constant exposure time of 300 ms was used for all measurements.

### 2.9 *E. coli*-Expressing RFP

Fluorescently labeled (RFP/chloramphenicol-resistant (CH)) *E. coli* was prepared using the BBa_J04450 plasmid encoding an RFP gene for constitutive expression. The plasmid was resuspended by pipetting 10 μL of molecular biology grade water into the designated well of the iGEM kit. After ∼5 min, 2 μL of the resuspended plasmid was transformed into 100 μL of *E. coli* One Shot TOP10 chemically competent cells suspension containing approximately 5 × 10^8^ cells. Transformation was performed using a standard heat shock protocol including the following incubations: 0.5 h on ice, 45 s in 42 °C wet bath, 2 min on ice, and 1 h recovery at 37 °C with 250 rpm shaking in 1 mL-rich LB medium (10 g L^–1^ Bacto tryptone, 5 g L^–1^ yeast extract, 10 g L^–1^ NaCl) supplemented with 10 mM MgCl_2_, 10 mM MgSO_4_, and 20 mM glucose. After recovery, cells were pelleted by pulsed centrifugation (∼1 min at up to 10 krpm), and approximately 900 μL of the supernatant was discarded. The cell pellet was resuspended in the remaining ∼100 μL of rich LB and plated on LB-Agar plates (LB recipe as described above, with additional 15 g agar per 1 L LB) containing 12.5 μg mL^–1^ CM. After overnight incubation at 37 °C, single pink colonies were selected and grown overnight in LB with 12.5 μg mL^–1^ CM at 37 °C and 250 rpm shaking.

### 2.10 Bacteria Preparation

GFP/Amp *E. coli* (K-12), RFP/CH *E. coli*, and non-fluorescent *E. coli* (K-12) were cultured from a single colony overnight in LB medium (with 100 μg mL^–1^ ampicillin, 12.5 μg mL^–1^ CM or without any antibiotics, respectively). 2 mL of bacteria suspension was diluted into 18 mL of PBS and centrifuged at 3260×*g* for 10 min. The pellet was suspended in PBS to reach an optical density at 600 nm (OD_600nm_) of 0.75 corresponding to 6 × 10^8^ cell mL^–1^ (or OD_600nm_ = 1.5 corresponding to 1.2 × 10^9^ cell mL^–1^ for heterogeneous culture experiment). GFP/Str *L. innocua* was cultured from a single colony overnight in BH medium with 100 μg mL^–1^ streptomycin and diluted to 1.2 × 10^9^ cell mL^–1^ as described above.

### 2.11 Binding Assay

Antibody-functionalized HNTs were suspended in PBS and mixed with the bacterial suspension to a final mixture of 2 mg particles mL^–1^ and 1 × 10^8^ cell mL^–1^ (of each bacterium). Control samples included a bacterial mixture with pristine HNTs, carboxylated E-HNTs, and neat bacteria suspension (no HNTs). Mixtures of bacteria and particles were gently shaken at 200 rpm for 2 h at RT in the dark to maintain optimal fluorescence and avoid potential light induced cytotoxicity of HNTs.^49^ Subsequently, samples were analyzed under a fluorescence microscope and by a high-throughput imaging flow cytometer ImageStreamX Mark II (Amnis) instrument (US).

#### Fluorescence Microscopy

0.6 μL of sample was gently mounted on clean silicon wafer and encircled with 25 μL of immersion oil. A coverslip (18 × 18 mm) was slowly laid on the drop minimizing shear stress. Samples were observed using a ZEISS Axio Scope A1 instrument.

#### High-Throughput Flow Cytometry

Immediately, after 2 h of gentle shaking, 25 μL of sample was drawn into the flow cytometer instrument, and the flow rate was automatically adjusted. Images were taken using the ×60 objective for bright field and green fluorescence channels. Every sample was prepared and run thrice analyzing the data using the IDEAS software (US). Raw images were gated for pixel intensity (focused images) and green fluorescence; at least 3000 gated images were collected for each experiment. “Bound” bacteria were distinguished based on the ratio between the bright field object area to bright field object perimeter. The “bound” population region on the plain of object area versus object perimeter was set manually based on the automatically suggested location. For further validation, the identified “bound” bacteria were visually inspected. The content of the “bound” bacteria was defined as the number of bound bacterial images divided by the total number of green fluorescence images.

### 2.12 Scanning Electron Microscopy

Mixtures of antibody-functionalized HNTs were fixed in glutaraldehyde solution (2.0% in PBS) overnight at 4 °C. Samples were subsequently washed in PBS and dehydrated by a series of ethanol in water solutions of 10, 25, 50, and 75% v/v (30 min incubation in each solution). Finally, the sample suspension in ethanol was mounted on a clean piece of silicon wafer and air-dried. A Carl Zeiss Ultra Plus (Germany) high-resolution scanning electron microscope was used at an accelerating voltage of 1.3 keV and a working distance of 3 mm.

### 2.13 Transmission Electron Microscopy and Energy-Dispersive X-ray Spectroscopy

Pristine and E-HNTs were mounted on a carbon type-B grid and imaged using an FEI Tecnai G2 T20 S-Twin transmission electron microscope coupled with an energy-dispersive X-ray (EDX) detector at an accelerating voltage of 200 keV. EDX results were processed using TIA (TEM Imaging & Analysis) software version 4.12, FEI Company, OR, USA.

### 2.14 X-ray Diffraction

X-ray diffractograms were collected using a Rigaku X-ray diffractometer (SmartLab, Rigaku, Tokyo, Japan) in the 2Θ range of 5°–90° at a rate of 2° per minute. The power of the Cu Kα radiation source was set to 40 kV, and the current was 30 mA.

### 2.15 Plate Count

Suspensions of GFP/Amp *E. coli* with/without particles (antibody-functionalized HNTs and carboxylated HNTs) were prepared as described for the binding assays (2 mg particles mL^–1^ and 1 × 10^8^ cell mL^–1^). After 2 h of gentle shaking, each sample was diluted with PBS by the decimal series: ×10^–5^, ×10^–6^, and ×10^–7^. 100 μL of each dilution was seeded on an LB agar plate, and colonies were counted after an overnight incubation at 37 °C. Each sample was prepared thrice, and each dilution was seeded twice.

### 2.16 Live/Dead Cell Staining

The biocompatibility of synthesized conjugates was also assessed according to their membrane-disruptive effect. A LIVE/DEAD BacLight bacterial viability kit was used to stain all bacteria cells with SYTO 9 (green fluorescence), whereas only the damaged membrane bacteria were stained with propidium iodide (red fluorescence). Suspensions of non-fluorescent *E. coli* with/without particles (antibody-functionalized HNTs and carboxylated HNTs) were prepared as described for the affinity assay (2 mg particles mL^–1^ and 1 × 10^8^ cell mL^–1^). Immediately after 2 h of gentle shaking, samples were vortexed and incubated with 10 μM of SYTO 9 and 60 μM of propidium iodide at RT in the dark for 15 min. Then, samples were vortexed again, and 5 μL of each stained sample was trapped between a clean silicon wafer and an 18 × 18 mm coverslip. Six images were taken for each sample using a fluorescence microscope equipped with ×20 dry objective. A constant exposure time of 600 ms was used for all measurements. Images were analyzed with ImageJ software. An additional sampling and staining step was performed after an overnight interval of gentle shaking.

## 3 Results and Discussion

### 3.1 Functionalization of HNTs with Oriented Anti *E. coli* Antibody

**Figure 1:**
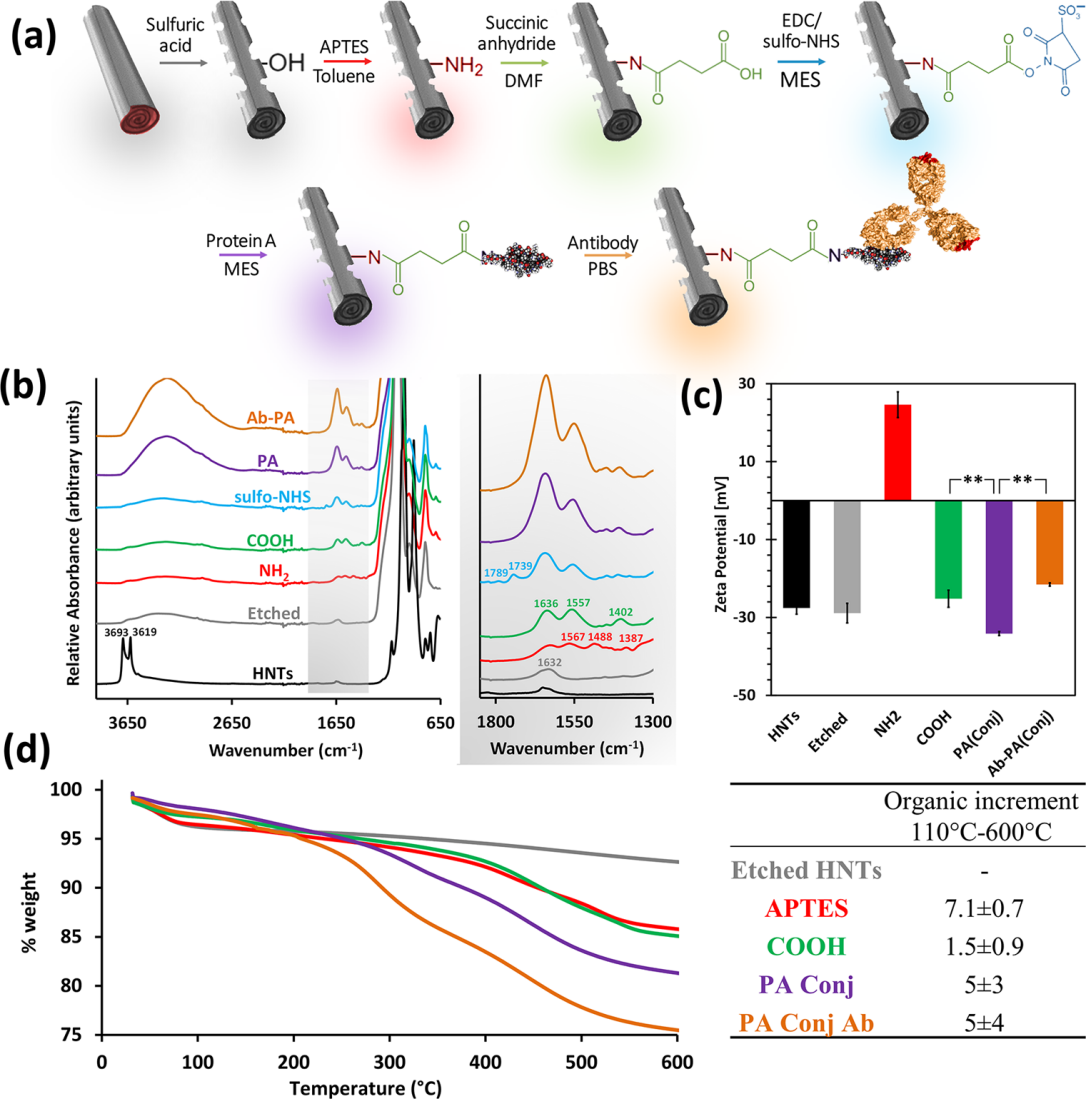
Synthetic steps followed for the oriented immobilization of the antibody onto the HNTs. (a) Schematic illustration of the synthesis procedure. (b) ATR-FTIR spectra of the HNTs following each of the synthetic steps (the spectra of the respective controls are included in Figure S1a, Supporting Information); all spectra are normalized to the inert highest peak. Right panel presents zoom-in spectra at a wavenumber range of 1800–1300 cm^–1^. Black trace for pristine HNTs, gray trace for E-HNTs, red trace for silanized HNTs (NH_2_), green trace for carboxylated HNTs (COOH), blue trace for sulfo-NHS-activated HNTs (sulfo-NHS), purple trace for PA-conjugated HNTs (PA), and orange trace for the antibody-immobilized HNTs (Ab-PA). (c) Zeta potential measurements of the HNTs following the different synthetic steps. **p < 0.01, one-tail *t*-test. Zeta potential values of the respective controls are included in Figure S1b, Supporting Information. (d) TGA measurements and the respective organic increment between each pair of successive steps (color scheme similar to all other results in this figure). The respective controls and derivative thermograms are included in Figure S2, Supporting Information. Figure 1a was created with graphics reproduced from ref (2). Copyright (2016) Wiley,^51^ Copyright (2010) Klein, Bjorkman https://creativecommons.org/licenses/by/4.0/, and^52^ Copyright (2013) Elsevier.

The synthetic route for immobilizing the antibody onto the HNTs is schematically outlined in Figure 1a. The first step includes acid etching of pristine HNTs to selectively dissolve the alumina layer leaving only a porous rolled silica layer which is also rough and perforated.^46^ The benefits of the etching process include an increase in specific surface area (by 4–5 folds, according to Brunauer–Emmett–Teller theory as previously reported^46^), lumen volume, overall porosity, and the number of surface reactive hydroxyl groups.^36−38,46^ Indeed, ATR-FTIR spectroscopy (Figure 1b) shows that the sharp peaks at 3693 and 3619 cm^–1^, which are characteristic of the aluminol groups on the pristine tube inner and interlayer surfaces, respectively,^50^ have diminished upon etching.

**Figure 2:**
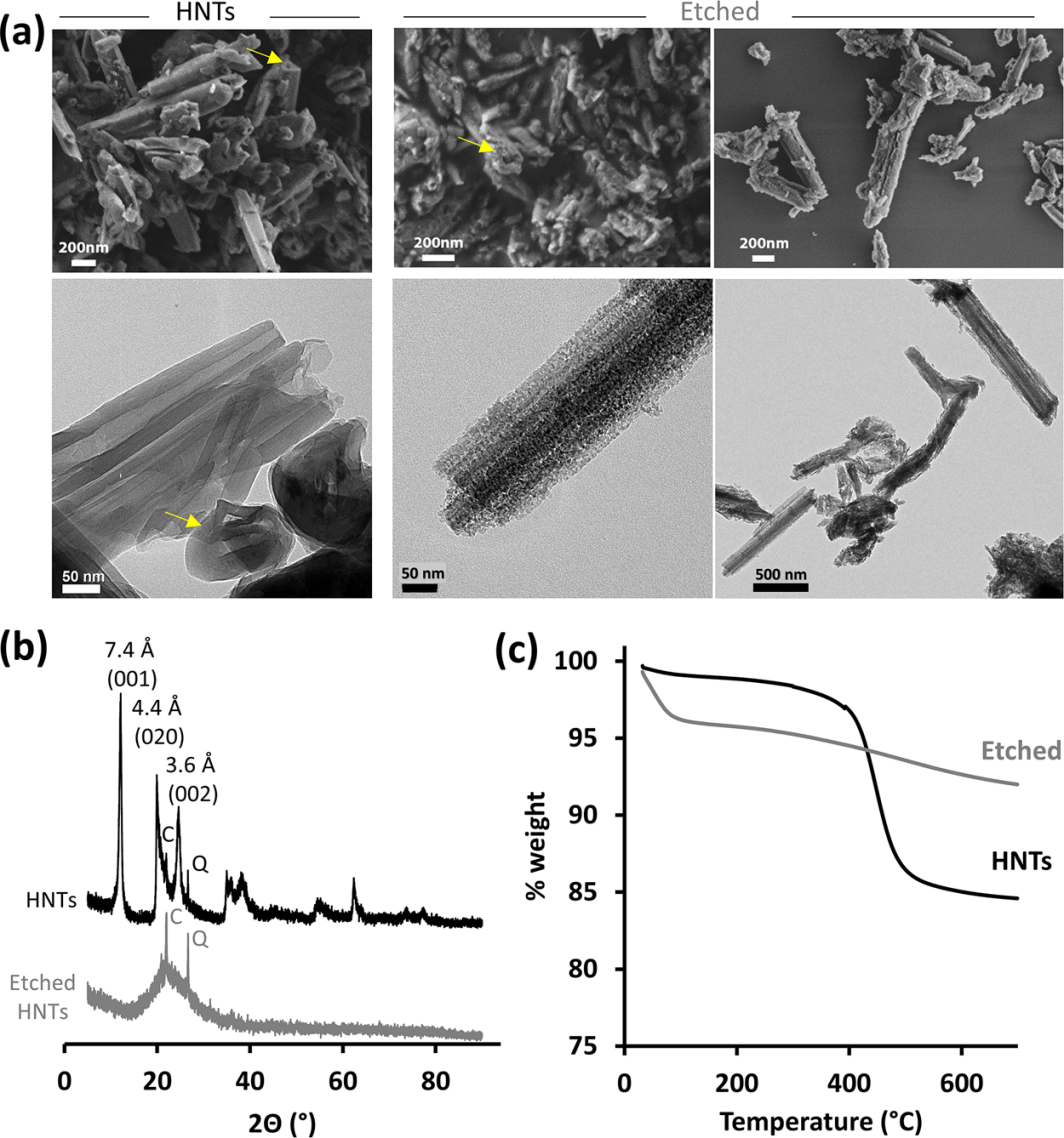
Characterization of E-HNTs. (a) Electron microscopy images of pristine HNTs (left) and E-HNTs (right) by HR-SEM (upper panel) and TEM (bottom panel). (b) X-ray diffractograms of pristine and E-HNTs. C—cristobalite,^53^ Q—quartz.^53^ (c) TGA of pristine and E-HNTs.

Figure 2 presents the nanostructure and the main characteristics of the E-HNTs in comparison to pristine HNTs. Scanning electron microscopy (SEM) images (see Figure 2a, upper panel) of the E-HNTs show that they exhibit a porous rodlike morphology (as indicated by the arrows in the micrographs which mark the pore opening), and their surface has roughened. Transmission electron microscopy (TEM) images (Figure 2a, lower panel) reveal that following etching the rather smooth surface of the pristine nanotubes becomes rough and granulated, while the elongated morphology of the particles is maintained. X-ray measurements (Figure 2b) display the characteristic peaks of pristine HNTs,^53^ whereas an amorphous halo is observed for the E-HNTs. These results suggest that the small granules observed by TEM are mostly amorphous silica and are in excellent agreement with previous report.^50^Figure 2c displays TGA thermograms of pristine and E-HNTs up to 600 °C. The weight loss observed up to 110 °C is ascribed to the loss of adsorbed water^39,54^ and is more pronounced for the E-HNTs, indicative of a higher surface area (note that both samples were stored under similar conditions). The second weight loss around 457 °C, which is ascribed to aluminol dihydroxylation,^39,55^ diminishes following etching and indicates that the majority of the alumina is removed. This is further supported by EDX-TEM measurements of the E-HNTs (Figure S3, Supporting Information) showing a low ratio of Al to Si (0.03) compared to the characteristic value of pristine HNTs (≥1).^4,46^ Thus, we can conclude that the etching process results in a high degree of conversion,^50^ namely, considerable alumina elimination and destruction of the layered lattice structure, while a porous rodlike structure is obtained.

After etching, the next step in the synthetic route is the amino-silanization of the E-HNTs using APTES,^39^ as outlined in Figure 1a. The amine residues on the silanized HNTs are converted by succinic anhydride in DMF to a hydrophilic carboxylic surface,^40^ after which a two-step EDC/sulfo-NHS reaction is performed to activate the surface for the subsequent PA conjugation.^27,54^ Finally, the antibody is immobilized onto the protein to allow its proper orientation, where the Fab fragments are free to interact with their target.^35^

Figure 1b presents the ATR-FTIR spectra of the HNTs following each of the synthetic steps. Following silanization, the HNTs exhibit typical peaks at 1567, 1488, and 1387 cm^–1^ corresponding to amine deformation (scissoring)^38^ and CH_2_ deformation vibration (scissoring and wagging, respectively^39^). The subsequent reaction with succinic anhydride yields peaks at 1636, 1557, and 1402 cm^–1^ which are ascribed to the carbonyl of an amide bond (amide I and amide II) and stretching vibration of a carboxyl residue, respectively.^56,57^ Successful covalent sulfo-NHS activation of the HNTs is confirmed by the characteristic peaks of cyclic imides detected at 1789 and 1739 cm^–1^.^56,57^ When the EDC/sulfo-NHS reaction is carried out on E-HNTs (no silanization and carboxylation), these two peaks are not observed, ruling out physical adsorption of the reactants on the HNTs’ surface (light blue trace in Figure S1a, Supporting Information). Succinimide-related peaks diminish after the conjugation of PA and amide I and amide II becomes more distinct for the antibody-immobilized HNTs via conjugated PA (referred to as Ab-PA_Conj_-HNTs). The amide peaks, attributed to the protein moieties on the HNTs, are significantly more distinct for PA conjugates (both PA_Conj_-HNTs and Ab-PA_Conj_-HNTs) in comparison to the respective controls, where PA was adsorbed on the particle surface (see the right panel of Figure S1a, Supporting Information for clarity). Hence, these results confirm the successful immobilization of the antibody via the conjugated PA.

The silanization and carboxylation reactions are further monitored by zeta potential measurements^17^ in water, and the results are summarized in Figure 1c. Both the neat and the E-HNTs exhibit a characteristic negative value of ∼−28 mV,^58^ while, following silanization, the modified HNTs are characterized by a positive zeta potential value of +25 ± 3 mV (attributed to the positively charged amino residues^59^). Subsequent carboxylation results in the inversion of the charge back to a negative value of −25 ± 2 mV.^40^ The same trend was observed for zeta potential measured in PBS (0.1 M pH 7.2) for E-HNTs before and after amino-salinization and carboxylation (see Table S2, Supporting Information). PA, whether conjugated (−34 ± 1 mV) or adsorbed (−38 ± 2 mV, see Figure S1b, Supporting Information), contributes similarly to the negative zeta potential value, possibly due to its negative charge in water (pI = 5.1^60^). The antibody immobilization onto conjugated PA is evidenced by a significant increase in zeta potential to a value of −21.5 ± 0.4 mV (*p* < 0.01, one-tail *t*-test). However, for the control sample, where the PA is only adsorbed PA, this behavior is not observed and the zeta potential value remains unchanged (−41 ± 1 mV, see Figure S1b, Supporting Information). This behavior and the significant difference in the zeta potential values may be attributed to a decreased affinity of the antibody to the adsorbed PA, possibly due to its surface-induced denaturation, as well as to lower surface coverage.

In order to further investigate the surface chemical modifications, TGA is used to measure the organic moieties on the HNTs following each of the synthetic steps, and the thermograms are presented in Figure 1d. The first mass loss up to 110 °C is ascribed to the loss of adsorbed water, and the remaining mass at 110–600 °C is related to organic matter decomposition^39,54^ (see Figure S2 for the corresponding derivative curves and Table S1 for a summary of the different weight loss events). The amino-silanized E-HNTs are characterized by two weight loss events, one at 110–263 °C and another at 265–600 °C, which are related to the loss of hydrogen-bonded and covalently bonded APTES molecules, respectively.^39,61^ The overall measured organic increment for this step is ∼7 %wt, implying good surface coverage of amino residues and possibly some oligomerization of APTES molecules.^43^ Following carboxylation, the organic content is increased by 1.5 %wt, signifying the reaction of the succinic anhydride with some of the amine residues.^47,54^ For the protein conjugates, the complexity of the respective thermograms is better elucidated by their derivative (see Figure S2 and Table S1, Supporting Information) at a temperature range typical for protein degradation.^62^ Protein A conjugation and the subsequent antibody immobilization result in an organic content increase of 5 ± 3 and 5 ± 4 % wt, respectively. This is ascribed to the successful binding of antibody onto the surface-conjugated PA. On the other hand, for both controls (with and without antibody) in which PA was only adsorbed onto the HNT surface rather than conjugated, an insignificant increase in organic mass was observed compared to the carboxylated HNTs (light purple and light orange traces compared with the green trace in Figure S2, Supporting Information). These results further support the FTIR and zeta potential measurements, demonstrating the successful immobilization of the antibody onto the E-HNTs.

**Figure 3:**
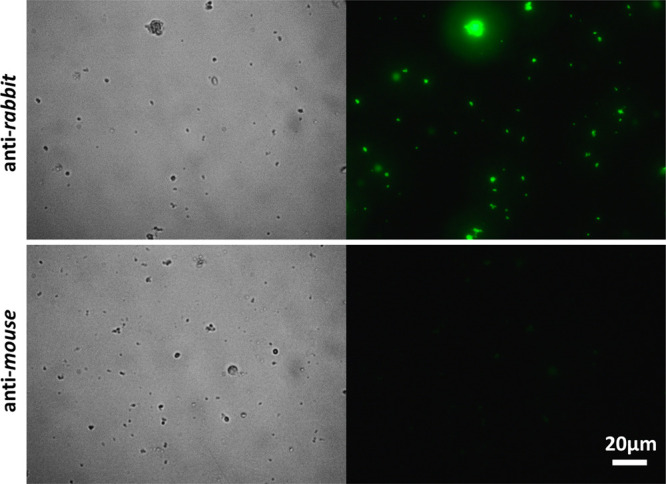
Bright field and corresponding fluorescence micrographs of antibody-functionalized HNTs following immuno-labeling with FITC-anti-rabbit antibody (upper panel) and FITC-anti-mouse antibody (lower panel).

The immobilization of the antibody (rabbit origin) is also investigated by fluorescent labeling with secondary anti-rabbit or mouse antibodies. Figure 3 presents fluorescence and bright field microscopy images of the antibody-functionalized HNTs following their incubation with fluorescent secondary antibodies. Fluorescence is only observed upon incubation of the HNTs with the anti-rabbit secondary antibody, while upon incubation with anti-mouse antibody, no fluorescence is detected. This confirms the successful immobilization of the antibody onto the particle surface and its antigenic activity. Upon incubation of the control carboxylated HNTs (not functionalized with PA and antibody) with the different secondary antibodies, no fluorescence is observed (see Figure S4, Supporting Information), ruling out non-specific adsorption of the labeled antibodies onto the HNTs.

### 3.2 Binding Assays

**Figure 4:**
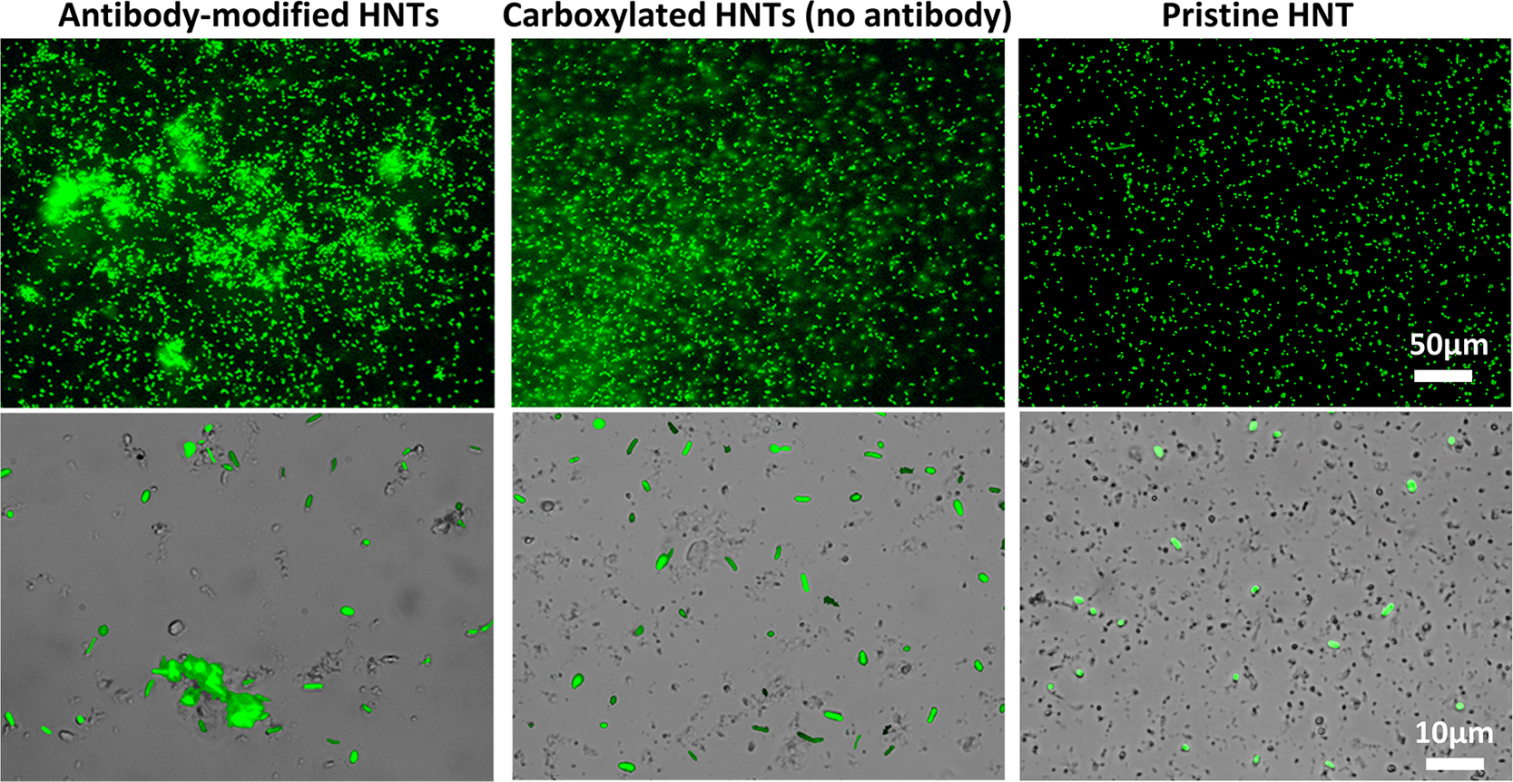
Green fluorescence and bright field micrographs for mixtures containing 1 × 10^8^ cell mL^–1^ of GFP-expressing *E. coli* (K-12) and 2 mg mL^–1^ of HNTs in PBS 0.1 M pH 7.2 after 2 h of gentle shaking at RT: Antibody-functionalized E-HNTs (left panel), carboxylated E-HNTs as control (no antibody, middle panel), and pristine HNTs (right panel).

The binding of the antibody-functionalized HNTs to their target bacteria is studied by fluorescence microscopy and high-throughput flow cytometry analysis. The HNTs (2 mg mL^–1^) were gently mixed with 1 × 10^8^ cell mL^–1^ of GFP-expressing *E. coli* (K-12) in PBS for 2 h, and samples were observed under a fluorescence microscope. Figure 4 depicts representative images of the suspensions, showing the formation of dense aggregates of cells and particles. These aggregates are not observed for the control carboxylated E-HNTs and pristine HNTs (see Figure 4 middle and right panels, respectively). Under higher magnification (Figure 4 lower panel), it is noticeable that the cells in the control samples are individually dispersed, while cells incubated with the functionalized HNTs tend to cluster at the proximity of the particles. These findings support the affinity between target bacteria and antibody-functionalized HNTs via antigenic recognition.

**Figure 5:**
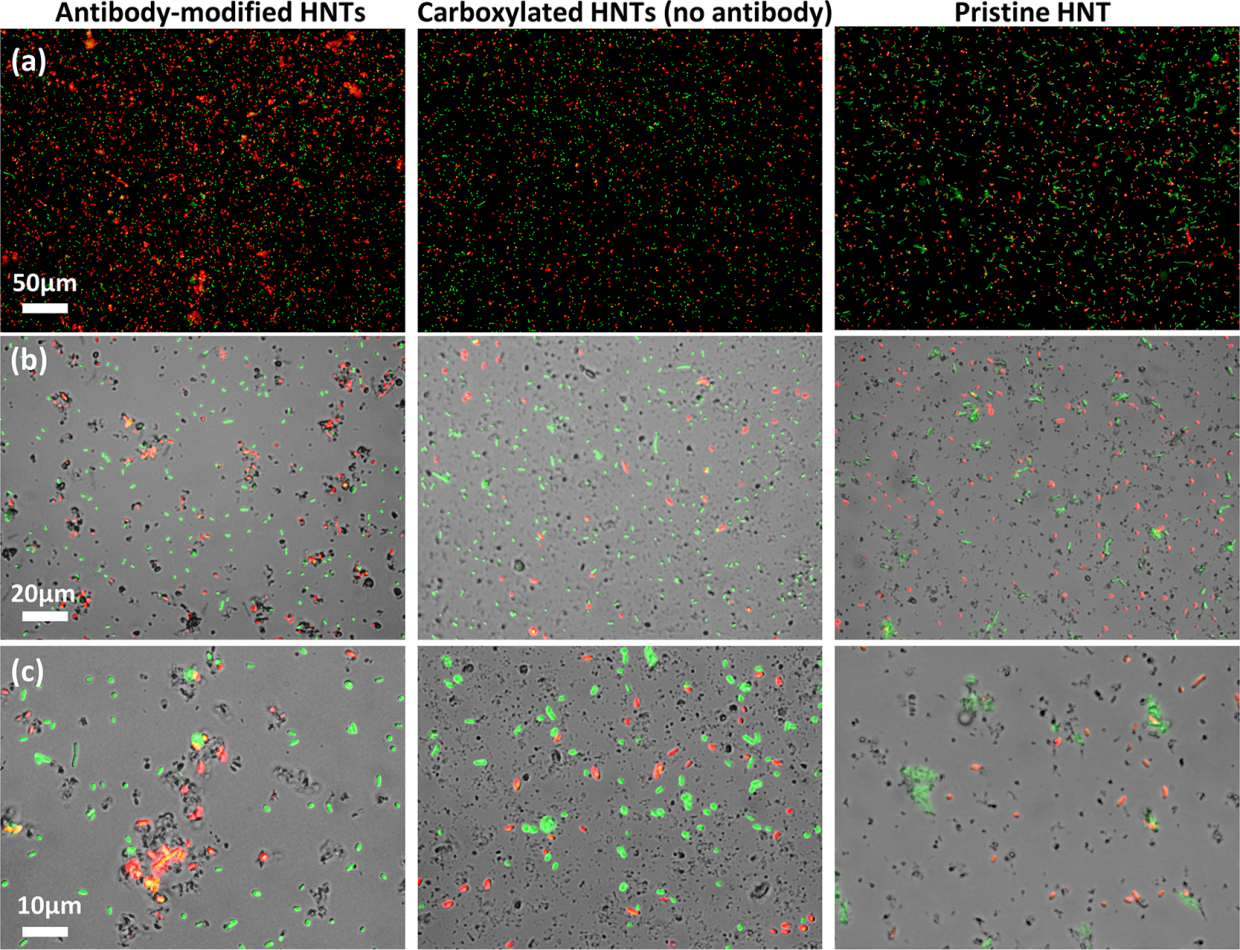
Green and red fluorescence micrographs of heterogeneous mixtures containing 1 × 10^8^ cell mL^–1^ of RFP-expressing *E. coli* (red), 1 × 10^8^ cell mL^–1^ of GFP-expressing *L. innocua* (green), and 2 mg mL^–1^ of HNTs in PBS 0.1 M pH 7.2 after 2 h of gentle shaking at RT: Antibody-functionalized carboxylated E-HNTs (left panel), carboxylated E-HNTs as control (no PA and antibody functionalization, middle panel), and pristine HNTs control (right panel). (a) Green and red fluorescence overlay; (b) bright field with green and red fluorescence overlay; and (c) bright field with green and red fluorescence overlay.

Next, the specificity of the modified HNTs against their target bacteria is investigated in a challenging heterogeneous culture containing 2 mg mL^–1^ HNTs, 1 × 10^8^ cell mL^–1^ of RFP-expressing *E. coli,* and 1 × 10^8^ cell mL^–1^ of GFP-expressing *L. innocua*. Representative images of the heterogeneous suspensions after 2 h of gentle shaking are presented in Figure 5. As observed in the homogenous culture (Figure 4), red *E. coli* cells form dense aggregates only in the presence of the functionalized HNTs (Figure 5, left panel) and not in the control samples containing the carboxylated E-HNTs or pristine HNTs (Figure 5, middle and right panels, respectively). Higher magnifications (middle and lower panels) reveal that the red *E. coli* cells and functionalized HNTs are selectively co-aggregated, whereas green *L. innocua* cells are excluded from the aggregates and remain individually dispersed. It is noteworthy that pristine (unmodified) HNTs are co-aggregated with the green *L. innocua* (non-target bacteria) rather than the target bacteria (red *E. coli*), as previously reported.^30^ The spontaneous interactions between unmodified HNTs and bacteria are ascribed to a combination of colloidal physical forces and biological factors, and as such they are expected to be unpredictable and unstable.^29^ Thus, the selective binding of the antibody-functionalized HNTs to the *E. coli*, in contrast to the nonspecific bacteria–clay interactions with pristine or carboxylated HNTs, confirms the activity of the anti *E. coli* antibodies immobilized onto the nanoclay particles.

**Figure 6:**
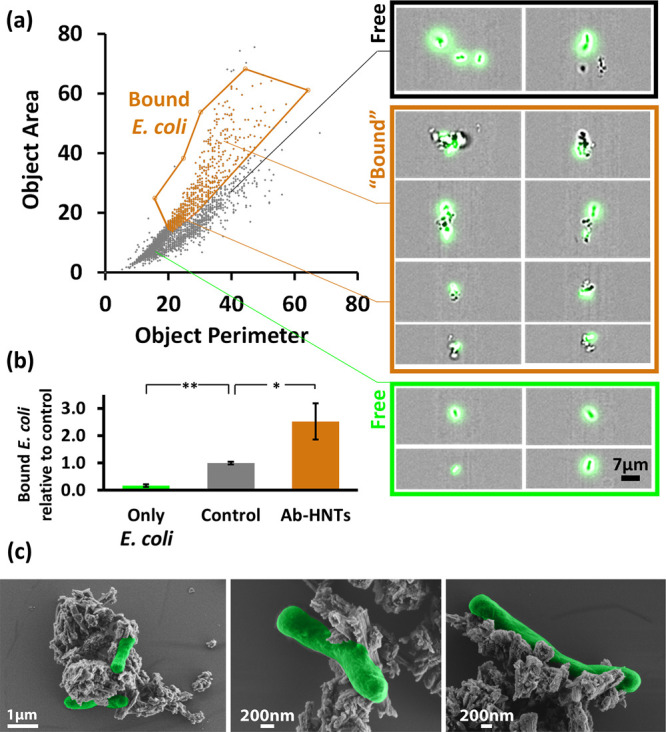
High-throughput flow cytometry measurements and HR-SEM images for mixtures of GFP/Amp *E. coli* (K-12, 1 × 10^8^ cell mL^–1^) and antibody-functionalized HNTs (2 mg mL^–1^) in PBS 0.1 M pH 7.2 after 2 h of gentle shaking at RT: (a) Left panel: a plot of the gated images for a typical single experiment positioned on the object area vs object perimeter plain with a well-defined region of “bound” bacteria (orange gate, all cytograms are provided in Figure S6, Supporting Information). Right panel: Representative images of “bound” vs “free” populations. (b) ImageStream quantification for content of bound bacteria for mixtures of *E. coli* with antibody-functionalized HNTs and with control HNTs (carboxylated). In addition, values for only *E. coli* suspensions are presented for comparison. Note that the content of “bound” bacteria is normalized to the HNT control. *p < 0.05, **p < 0.01, *n* = 3, one-tail *t*-test. (c) HR-SEM images of antibody-functionalized HNTs and *E. coli* mixtures. Bacterial cells are false colored in green for clarity.

In order to quantitatively study the affinity between the functionalized HNTs and the cells, we employ high-throughput flow cytometry analysis. The particles and the bacterial suspensions (prepared as previously described for homogenous culture) are introduced into the instrument and are analyzed under flow conditions. Each particulate object is separately photographed using ×60 objective for bright field and green fluorescence channels. Analysis is performed on images with green fluorescence that are well focused narrowing to at least 3000 gated images per experiment. Images of “bound” bacteria are distinguishable from “free” bacteria based on the shape of the bright field object, where the “free” (individually dispersed cells) population is characterized by a distinct object area and object perimeter values. On the other hand, the “bound” bacteria population, in which the HNTs and the cells are co-aggregated, exhibits a statistically different set of values (see Figure S5 for a schematic illustration of the method concept and steps). Figure 6a presents a summary of the results of a characteristic single experiment in which using the IDEAS software (Amnis) images are plotted according to the object area versus the object perimeter. The orange region in Figure 6a represents the “bound” population and is set semi-automatically and kept constant for all subsequent experiments. Representative images (see the right panel of Figure 6a) depict the characteristic populations of “bound” bacteria versus “free” bacteria. The content of “bound” bacteria is calculated by dividing the number of images within the orange region to the total number of focused green fluorescence images (see Figure S6 for all cytograms). These measurements and analyses are performed for antibody-functionalized HNTs and bacteria suspensions as well as for non-functionalized HNTs (carboxylated) and bacteria mixtures. The results are summarized in Figure 6b and are compared to values collected for neat *E. coli* suspensions (no HNTs). The values in Figure 6b are normalized to the content of “bound” bacteria for non-functionalized HNTs. Suspensions of antibody-functionalized HNTs exhibit a significantly higher (>2.5 fold, *p* < 0.05, one-tail *t*-test, *n* = 3) content of “bound” bacteria in comparison to the control HNTs. This trend corresponds well with the fluorescence microscopy analysis (Figure 4) and clearly indicates the superior affinity of the antibody-functionalized HNTs toward the *E. coli* cells. It should be noted that possible false-positive contribution of free *E. coli* cells to the calculated content of “bound” bacteria is minimal (*p* < 0.01, one-tail *t*-test, *n* = 3) as shown by the values of the *E. coli* suspensions (no HNTs), see Figure 6b.

HR-SEM images for suspensions of antibody-functionalized HNTs and bacteria are presented in Figure 6c, revealing intact cells that are attached to aggregates of porous rodlike particles, with much similarity to the flow cytometry images. We conclude that bacteria–particle interactions are indeed antibody mediated since no elongated filaments of the polysaccharide biofilm matrix is observed in HR-SEM images.^63−66^

### 3.3 Viability Studies

There is no clear consensus regarding the cytotoxicity of pristine HNTs toward bacterial cells,^49,67−69^ and any chemical modification of these particles may alter their behavior.^70^ Thus, we investigate the effect of the synthesized antibody-functionalized HNTs on the viability of *E. coli*.

**Figure 7:**
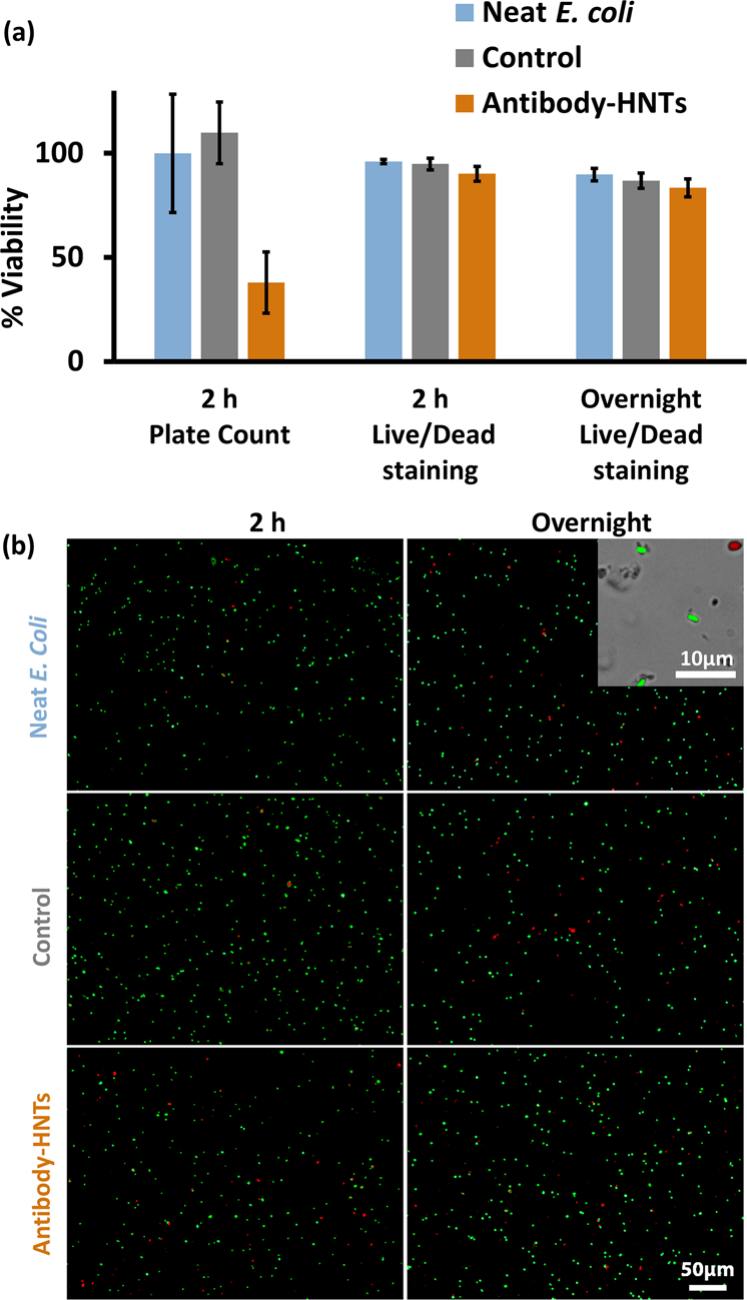
Viability of *E. coli* (K-12, 1 × 10^8^ cell mL^–1^) following a 2 h or overnight incubation with/without antibody-functionalized HNTs and control (carboxylated) HNTs (2 mg mL^–1^), as measured by plate count and live/dead cell staining. Incubation performed in PBS 0.1 M pH 7.2 under gentle shaking at RT. (a) Normalized viability results. Note that for plate count, viability is normalized to neat *E. coli* suspension. Viability values by live/dead staining are expressed as the percentage of green-stained bacteria from the total cells after 2 h and overnight incubation. (b) Representative fluorescent micrographs following live/dead cell staining. Inset shows a higher magnification image for an overlay of bright field with green and red fluorescence.

Mixtures of antibody-functionalized HNTs with bacteria are prepared as described in the previous section, and the cell viability is quantified by plate count and live/dead staining. Figure 7 summarizes the results obtained by the two techniques in comparison to the control HNTs (carboxylated) and neat *E. coli* suspensions.

The viability of the antibody-functionalized HNTs (35 ± 15%), by plate count, is substantially lower than that of both controls. Yet, the actual counted bacterial concentration for antibody-functionalized HNTs (after 2 h of incubation) is 0.6 ± 0.2 × 10^8^ cfu mL^–1^ which is comparable to the initial *E. coli* concentration (1 × 10^8^ cell/mL^–1^) in the mixture. During this 2 h incubation, the bacterial count for both controls increases to a value of ∼2 × 10^8^ cfu mL^–1^. Thus, these results suggest that the antibody-functionalized HNTs exert a bacteriostatic effect on *E. coli*. This is further supported by the results of the live/dead staining. Figure 7b presents characteristic fluorescent images of the stained cells, where the majority of the cells are stained in green for all suspensions. Only few red-stained (dead) cells are observed for both the antibody-functionalized and control HNTs, similar to the neat *E. coli* suspensions. The viability values by live/dead staining are expressed as the percentage of green-stained bacteria from the total cells and are presented in Figure 7a. Indeed, only a minor decrease in cell viability (to a value of 90 ± 4%) is observed for the antibody-functionalized HNTs after 2 h, and this trend is maintained following an overnight incubation. Our results are consistent with previous work,^69^ where viability values of ∼85% were measured for *E. coli* incubated with pristine HNTs (1.0 mg HNTs mL^–1^) for 8 h. The authors attributed this inhibitory effect to light-dependent oxidative stress,^49^ possibly inflicted by silica generated reactive oxygen species.^69^ Furthermore, protein adsorbed onto HNTs was found to increase their biocompatibility toward *E. coli* compared with unmodified HNTs.^69^

We suggest that a possible explanation for the discrepancy between plate count and live/dead viability values may be accounted for hindered cell division due to the binding of the bacteria to the antibody-functionalized HNTs.

## 4 Conclusions

This work constitutes the first demonstration of successful selective targeting of live bacterial cells by suspended HNTs functionalized with an oriented antibody. Oriented immobilization is realized through interaction of the antibody with PA conjugated to carboxylated HNTs via a facile two-step EDC/sulfo-NHS reaction. The modified HNTs exhibit superior binding toward target bacteria (*E. coli*) in comparison to the non-modified control, as quantitatively assessed by high-throughput flow cytometry (ImageStream). Moreover, selective binding of the target cells to the HNTs is demonstrated in a heterogeneous culture containing *E. coli* and *L. innocua*. Bound *E. coli* cells maintain 83% viability, even after overnight incubation, suggesting the biocompatibility of these HNT hybrids. The approach presented here is highly generic and can be easily adapted to target other microorganisms. Thus, this proof-of-concept work can be potentially employed for selective manipulation and delivery of payloads to target cells utilizing the advantageous characteristics of natural clay HNTs and their unique interactions with biological cells.^29^

## Supporting Information Available

Surface modification of HNTs: background and details; characterization of the different synthetic steps followed for the oriented immobilization of the antibody onto the HNTs; TGA; TEM EDX measurement of etched HNTs; flow chart for the analytical procedure of high-throughput flow cytometry analysis; and high-throughput flow cytometry measurements (PDF)

## ABBREVIATIONS

Ab-PA_Ad_-HNTs: carboxylated etched HNTs with adsorbed protein A incubated with antibody
Ab-PA_Conj_-HNTs: protein A conjugated etched HNTs with immobilized antibody
APTES: 3-aminopropyltriethoxysilane
ATR-FTIR: attenuated total reflectance Fourier transform-infrared
BSA: bovine serum albumin
cfu: colony forming units
DMF: dimethylformamide
*E. coli*: *Escherichia coli*
EDC: (1-ethyl-3-(3-dimethylaminopropyl)carbodiimide)
EDX: energy-dispersive X-ray spectroscopy
E-HNTs: etched HNTs
FITC: fluorescein isothiocyanate
GFP: green fluorescent protein
HNTs: halloysite nanotubes
HR-SEM: high-resolution scanning electron microscopy
LB: Luria broth
*L. innocua*: *Listeria innocua*
MES: 2-(*N*-morpholino)ethanesulfonic acid
OD_600_: optical density at 600 nm
PA: protein A
PA_Ad_-HNTs: carboxylated etched HNTs with adsorbed protein A
PA_Conj_-HNTs: protein A conjugated etched HNTs
PBS: phosphate buffer saline
RFP: red fluorescent protein
RT: room temperature
sulfo-NHS: *N*-hydroxysulfosuccinimide
TEM: transmission electron microscopy
TGA: thermal gravimetric analysis

## References

[ref1] Churchman G. J.; Pasbakhsh P.; Hillier S. The Rise and Rise of Halloysite. Clay Miner. 2016, 51, 303–308. 10.1180/claymin.2016.051.3.00.

[ref2] Lvov Y.; Wang W.; Zhang L.; Fakhrullin R. Halloysite Clay Nanotubes for Loading and Sustained Release of Functional Compounds. Adv. Mater. 2016, 28, 1227–1250. 10.1002/adma.201502341. 26438998

[ref3] Joussein E. Geology and Mineralogy of Nanosized Tubular Halloysite. In Nanosized Tubular Clay Minerals - Halloysite and Imogolite; Peng Yuan A. T., Faïza B., Eds.; Elsevier, 2016, Chapter 2, pp 12–48.

[ref4] Yang H.; Zhang Y.; Ouyang J. Physicochemical Properties of Halloysite. In Nanosized Tubular Clay Minerals - Halloysite and Imogolite; Peng Yuan A. T., Faïza B., Eds.; Elsevier, 2016, Chapter 4, pp 67–91.

[ref5] Wang X.; Gong J.; Rong R.; Gui Z.; Hu T.; Xu X. Halloysite Nanotubes-induced Al Accumulation and Fibrotic Response in Lung of Mice after 30-day Repeated Oral Administration. J. Agric. Food Chem. 2018, 66, 2925–2933. 10.1021/acs.jafc.7b04615. 29470912

[ref6] Long Z.; Wu Y.-P.; Gao H.-Y.; Zhang J.; Ou X.; He R.-R.; Liu M. In vitro and in vivo toxicity evaluation of halloysite nanotubes. J. Mater. Chem. B 2018, 6, 7204–7216. 10.1039/c8tb01382a. 32254633

[ref7] Fakhrullina G. I.; Akhatova F. S.; Lvov Y. M.; Fakhrullin R. F. Toxicity of Halloysite Clay Nanotubes In Vivo: A Caenorhabditis Elegans Study. Environ. Sci.: Nano 2015, 2, 54–59. 10.1039/c4en00135d.

[ref8] Kryuchkova M.; Danilushkina A.; Lvov Y.; Fakhrullin R. Evaluation of Toxicity of Nanoclays and Graphene Oxide In Vivo: A Paramecium caudatum Study. Environ. Sci.: Nano 2016, 3, 442–452. 10.1039/c5en00201j.

[ref9] Maisanaba S.; Pichardo S.; Puerto M.; Gutiérrez-Praena D.; Cameán A. M.; Jos A. Toxicological Evaluation of Clay Minerals and Derived Nanocomposites: A Review. Environ. Res. 2015, 138, 233–254. 10.1016/j.envres.2014.12.024. 25732897

[ref10] Liu M.; Fakhrullin R.; Novikov A.; Panchal A.; Lvov Y. Tubule Nanoclay-organic Heterostructures for Biomedical Applications. Macromol. Biosci. 2019, 19, e1800419 10.1002/mabi.201800419. 30565394

[ref11] Massaro M.; Lazzara G.; Milioto S.; Noto R.; Riela S. Covalently Modified Halloysite Clay Nanotubes: Synthesis, Properties, Biological and Medical Applications. J. Mater. Chem. B 2017, 5, 2867–2882. 10.1039/c7tb00316a. 32263981

[ref12] Santos A. C.; Ferreira C.; Veiga F.; Ribeiro A. J.; Panchal A.; Lvov Y.; Agarwal A. Halloysite Clay Nanotubes for Life Sciences Applications: from Drug Encapsulation to Bioscaffold. Adv. Colloid Interface Sci. 2018, 257, 58–70. 10.1016/j.cis.2018.05.007. 29887382

[ref13] Satish S.; Tharmavaram M.; Rawtani D. Halloysite Nanotubes as a Nature’s Boon for Biomedical Applications. Nanobiomedicine 2019, 6, 1849543519863625 10.1177/1849543519863625. 31320940 PMC6628522

[ref14] Stavitskaya A.; Batasheva S.; Vinokurov V.; Fakhrullina G.; Sangarov V.; Lvov Y.; Fakhrullin R. Antimicrobial Applications of Clay Nanotube-based Composites. Nanomaterials 2019, 9, 708 10.3390/nano9050708. PMC6567215 31067741

[ref15] Massaro M.; Campofelice A.; Colletti C. G.; Lazzara G.; Noto R.; Riela S. Functionalized Halloysite Nanotubes: Efficient Carrier Systems for Antifungine drugs. Appl. Clay Sci. 2018, 160, 186–192. 10.1016/j.clay.2018.01.005.

[ref16] Lvov Y. M.; DeVilliers M. M.; Fakhrullin R. F. The Application of Halloysite Tubule Nanoclay in Drug Delivery. Expert Opin. Drug Delivery 2016, 13, 977–986. 10.1517/17425247.2016.1169271. 27027933

[ref17] Tharmavaram M.; Pandey G.; Rawtani D. Surface Modified Halloysite Nanotubes: A Flexible Interface for Biological, Environmental and Catalytic Applications. Adv. Colloid Interface Sci. 2018, 261, 82–101. 10.1016/j.cis.2018.09.001. 30243667

[ref18] Price R. R.; Gaber B. P.; Lvov Y. In-vitro release characteristics of tetracycline HCl, khellin and nicotinamide adenine dineculeotide from halloysite; a cylindrical mineral. J. Microencapsulation 2001, 18, 713–722. 10.1080/02652040010019532. 11695636

[ref19] Mills D.; Lvov Y. Ceramic Nanotube Composites with Sustained Drug Release Capability for Implants, Bone Repair and Regenaration. U.S. Patent 9,192,912 B1, November 24, 2015.

[ref20] Zhou T.; Jia L.; Luo Y. F.; Xu J.; Chen R. H.; Ge Z. J.; Ma T. L.; Chen H.; Zhu T. F. Multifunctional Nanocomposite Based on Halloysite Nanotubes for Efficient Luminescent Bioimaging and Magnetic Resonance Imaging. Int. J. Nanomed. 2016, 11, 4765–4776. 10.2147/IJN.S110081. PMC5034929 27698562

[ref21] Li X.; Chen J.; Liu H.; Deng Z.; Li J.; Ren T.; Huang L.; Chen W.; Yang Y.; Zhong S. beta-Cyclodextrin Coated and Folic Acid Conjugated Magnetic Halloysite Nanotubes for Targeting and Isolating of Cancer Cells. Colloids Surf., B 2019, 181, 379–388. 10.1016/j.colsurfb.2019.05.068. 31170644

[ref22] Wu Y.-P.; Yang J.; Gao H.-Y.; Shen Y.; Jiang L.; Zhou C.; Li Y.-F.; He R.-R.; Liu M. Folate-conjugated Halloysite Nanotubes, an Efficient Drug Carrier, Deliver Doxorubicin for Targeted Therapy of Breast Cancer. ACS Appl. Nano Mater. 2018, 1, 595–608. 10.1021/acsanm.7b00087.

[ref23] Yamina A. M.; Fizir M.; Itatahine A.; He H.; Dramou P. Preparation of Multifunctional PEG-graft-halloysite Nanotubes for Controlled Drug Release, Tumor Cell Targeting, and Bio-imaging. Colloids Surf., B 2018, 170, 322–329. 10.1016/j.colsurfb.2018.06.042. 29936385

[ref24] Dzamukova M. R.; Naumenko E. A.; Lvov Y. M.; Fakhrullin R. F. Enzyme-activated Intracellular Drug Delivery with Tubule Clay Nanoformulation. Sci. Rep. 2015, 5, 10560 10.1038/srep10560. 25976444 PMC4432568

[ref25] Fakhrullina G.; Khakimova E.; Akhatova F.; Lazzara G.; Parisi F.; Fakhrullin R. Selective Antimicrobial Effects of Curcumin@Halloysite Nanoformulation: A Caenorhabditis elegans Study. ACS Appl. Mater. Interfaces 2019, 11, 23050–23064. 10.1021/acsami.9b07499. 31180643

[ref26] Hughes A. D.; Mattison J.; Western L. T.; Powderly J. D.; Greene B. T.; King M. R. Microtube Device for Selectin-mediated Capture of Viable Circulating Tumor Cells from Blood. Clin. Chem. 2012, 58, 846–853. 10.1373/clinchem.2011.176669. 22344286

[ref27] He R.; Liu M.; Shen Y.; Liang R.; Liu W.; Zhou C. Simple Fabrication of Rough Halloysite Nanotubes Coatings by Thermal Spraying for High Performance Tumor Cells Capture. Mater. Sci. Eng., C 2018, 85, 170–181. 10.1016/j.msec.2017.12.030. 29407145

[ref28] Konnova S. A.; Sharipova I. R.; Demina T. A.; Osin Y. N.; Yarullina D. R.; Ilinskaya O. N.; Lvov Y. M.; Fakhrullin R. F. Biomimetic Cell-mediated Three-dimensional Assembly of Halloysite Nanotubes. Chem. Commun. 2013, 49, 4208 10.1039/c2cc38254g. 23292434

[ref29] Prinz Setter O.; Segal E. Halloysite nanotubes - the nano-bio interface. Nanoscale 2020, 12, 23444–23460. 10.1039/d0nr06820a. 33237090

[ref30] Barr M. Adsorption Studies on Clays II. The Adsorption of Bacteria by Activated Attapulgite, Halloysite, and Kaolin. J. Am. Pharm. Assoc. 1957, 46, 490–492. 10.1002/jps.3030460810. 13491430

[ref31] Tan D.; Zhang H.; Sun S.; Dong F.; Sun H.; Li B. Rapid Flocculation-sedimentation of Microalgae with Organosilane-functionalized Halloysite. Appl. Clay Sci. 2019, 177, 37–42. 10.1016/j.clay.2019.05.005.

[ref32] Konnova S. A.; Lvov Y. M.; Fakhrullin R. F. Magnetic Halloysite Nanotubes for Yeast Cell Surface Engineering. Clay Miner. 2016, 51, 429–433. 10.1180/claymin.2016.051.3.07.

[ref33] Partovinia A.; Koosha M. Fabrication of Novel Nanocomposite Nanofibrous Matrices Retaining High Concentration of Microbial Cells for Heavy Crude Oil Biodegradation. eXPRESS Polym. Lett. 2019, 13, 484–499. 10.3144/expresspolymlett.2019.40.

[ref34] Yu T.; Swientoniewski L. T.; Omarova M.; Li M.-C.; Negulescu II; Jiang N.; Darvish O. A.; Panchal A.; Blake D. A.; Wu Q.; Lvov Y. M.; John V. T.; Zhang D. Investigation of Amphiphilic Polypeptoid-functionalized Halloysite Nanotubes as Emulsion Stabilizer for Oil Spill Remediation. ACS Appl. Mater. Interfaces 2019, 11, 27944–27953. 10.1021/acsami.9b08623. 31306577

[ref35] Boyle M. D. P.; Reis K. J. Bacterial Fc Receptors. Nat. Biotechnol. 1987, 5, 697–703. 10.1038/nbt0787-697.

[ref36] Belkassa K.; Bessaha F.; Marouf-Khelifa K.; Batonneau-Gener I.; Comparot J.-d.; Khelifa A. Physicochemical and Adsorptive Properties of A Heat-treated and Acid-leached Algerian Halloysite. Colloids Surf., A 2013, 421, 26–33. 10.1016/j.colsurfa.2012.12.048.

[ref37] Garcia-Garcia D.; Ferri J. M.; Ripoll L.; Hidalgo M.; Lopez-Martinez J.; Balart R. Characterization of Selectively Etched Halloysite Nanotubes by Acid Treatment. Appl. Surf. Sci. 2017, 422, 616–625. 10.1016/j.apsusc.2017.06.104.

[ref38] Sun P.; Liu G.; Lv D.; Dong X.; Wu J.; Wang D. Effective Activation of Halloysite Nanotubes by Piranha Solution for Amine Modification via Silane Coupling Chemistry. RSC Adv. 2015, 5, 52916–52925. 10.1039/c5ra04444h.

[ref39] Yuan P.; Southon P. D.; Liu Z.; Green M. E. R.; Hook J. M.; Antill S. J.; Kepert C. J. Functionalization of Halloysite Clay Nanotubes by Grafting with γ-aminopropyltriethoxysilane. J. Phys. Chem. C 2008, 112, 15742–15751. 10.1021/jp805657t.

[ref40] Joo Y.; Jeon Y.; Lee S. U.; Sim J. H.; Ryu J.; Lee S.; Lee H.; Sohn D. Aggregation and Stabilization of Sarboxylic Acid Functionalized Halloysite Nanotubes (HNT-COOH). J. Phys. Chem. C 2012, 116, 18230–18235. 10.1021/jp3038945.

[ref41] Hermanson G. T. Immobilization of Ligands on Chromatography Supports. In Bioconjugate Techniques, 3rd ed.; Hermanson G. T., Ed.; Academic Press: Boston, 2013, Chapter 15, pp 589–740.

[ref42] Arshavsky-Graham S.; Urmann K.; Salama R.; Massad-Ivanir N.; Walter J.-G.; Scheper T.; Segal E. Aptamers vs. antibodies as capture probes in optical porous silicon biosensors. Analyst 2020, 145, 4991–5003. 10.1039/d0an00178c. 32519701

[ref43] Demirel G.; Çaykara T.; Akaoğlu B.; Çakmak M. Construction of a novel multilayer system and its use for oriented immobilization of immunoglobulin G. Surf. Sci. 2007, 601, 4563–4570. 10.1016/j.susc.2007.06.034.

[ref44] Baptista P. V.; McCusker M. P.; Carvalho A.; Ferreira D. A.; Mohan N. M.; Martins M.; Fernandes A. R. Nano-strategies to Fight Multidrug Resistant Bacteria-“A Battle of the Titans”. Front. Microbiol. 2018, 9, 1441 10.3389/fmicb.2018.01441. 30013539 PMC6036605

[ref45] Morrison K. D.; Misra R.; Williams L. B. Unearthing the Antibacterial Mechanism of Medicinal Clay: A Geochemical Approach to Combating Antibiotic Resistance. Sci. Rep. 2016, 6, 19043 10.1038/srep19043. 26743034 PMC4705759

[ref46] Barfod K. K.; Bendtsen K. M.; Berthing T.; Koivisto A. J.; Poulsen S. S.; Segal E.; Verleysen E.; Mast J.; Holländer A.; Jensen K. A.; Hougaard K. S.; Vogel U. Increased Surface Area of Halloysite Nanotubes due to Surface Modification Predicts Lung Inflammation and Acute Phase Response after Pulmonary Exposure in Mice. Environ. Toxicol. Pharmacol. 2020, 73, 103266 10.1016/j.etap.2019.103266. 31707308

[ref47] Liu M.; Chang Y.; Yang J.; You Y.; He R.; Chen T.; Zhou C. Functionalized Halloysite Nanotube by Chitosan Grafting for Drug Delivery of Curcumin to Achieve Enhanced Anticancer Efficacy. J. Mater. Chem. B 2016, 4, 2253–2263. 10.1039/c5tb02725j. 32263221

[ref48] Staros J. V. N-hydroxysulfosuccinimide Active Esters: bis(N-hydroxysulfosuccinimide) Esters of Two Dicarboxylic Acids Are Hydrophilic, Membrane-impermeant, Protein Cross-linkers. Biochemistry 1982, 21, 3950–3955. 10.1021/bi00260a008. 7126526

[ref49] Taylor A. A.; Aron G. M.; Beall G. W.; Dharmasiri N.; Zhang Y.; McLean R. J. C. Carbon and Clay Nanoparticles Induce Minimal Stress Responses in Gram Negative Bacteria and Eukaryotic Fish Cells. Environ. Toxicol. 2014, 29, 961–968. 10.1002/tox.21824. 23125163

[ref50] Abdullayev E.; Joshi A.; Wei W.; Zhao Y.; Lvov Y. Enlargement of Halloysite Clay Nanotube Lumen by Selective Etching of Aluminum Oxide. ACS Nano 2012, 6, 7216–7226. 10.1021/nn302328x. 22838310

[ref51] Klein J. S.; Bjorkman P. J. Few and far between: how HIV may be evading antibody avidity. PLoS Pathog. 2010, 6, e1000908 10.1371/journal.ppat.1000908. 20523901 PMC2877745

[ref52] Hermanson G. T. Introduction to Bioconjugation. In Bioconjugate Techniques, 3rd ed.; Hermanson G. T., Ed.; Academic Press: Boston, 2013; pp 1–125.

[ref53] Pasbakhsh P.; Churchman G. J.; Keeling J. L. Characterisation of properties of various halloysites relevant to their use as nanotubes and microfibre fillers. Appl. Clay Sci. 2013, 74, 47–57. 10.1016/j.clay.2012.06.014.

[ref54] Zhao Q.; Liu C.; Liu J.; Zhang Y. Development of a Novel Polyethersulfone Ultrafiltration Membrane with Antibacterial Activity and High Flux Containing Halloysite Nanotubes Loaded with Lysozyme. RSC Adv. 2015, 5, 38646–38653. 10.1039/c5ra05062f.

[ref55] Daou I.; Lecomte-Nana G.; Tessier-Doyen N.; Peyratout C.; Gonon M.; Guinebretiere R. Probing the Dehydroxylation of Kaolinite and Halloysite by In Situ High Temperature X-ray Diffraction. Minerals 2020, 10, 480 10.3390/min10050480.

[ref56] Tenenbaum E.; Ben-Dov N.; Segal E. Tethered Lipid Bilayers within Porous Si Nanostructures: A Platform for (Optical) Real-time Monitoring of Membrane-associated Processes. Langmuir 2015, 31, 5244–5251. 10.1021/acs.langmuir.5b00935. 25902286

[ref57] Kim J.; Cho J.; Seidler P. M.; Kurland N. E.; Yadavalli V. K. Investigations of Chemical Modifications of Amino-terminated Organic Films on Silicon Substrates and Controlled Protein Immobilization. Langmuir 2010, 26, 2599–2608. 10.1021/la904027p. 20095550

[ref58] Vergaro V.; Abdullayev E.; Lvov Y. M.; Zeitoun A.; Cingolani R.; Rinaldi R.; Leporatti S. Cytocompatibility and Uptake of Hlloysite Clay Nanotubes. Biomacromolecules 2010, 11, 820–826. 10.1021/bm9014446. 20170093

[ref59] Shi Y.-F.; Tian Z.; Zhang Y.; Shen H.-B.; Jia N.-Q. Functionalized Halloysite Nanotube-based Carrier for Intracellular Delivery of Antisense Oligonucleotides. Nanoscale Res. Lett. 2011, 6, 608 10.1186/1556-276x-6-608. 22122822 PMC3236537

[ref60] Iangone J. J. Protein A of Staphylococcus aureus and Related Immunoglobulin Receptors Produced by Streptococci and Pneumonococci. In Advances in Immunology; Dixon F. J., Kunkel H. G., Eds.; Academic Press, 1982; pp 157–252. 7051784

[ref61] Zargarian S. S.; Haddadi-Asl V.; Hematpour H. Carboxylic acid functionalization of halloysite nanotubes for sustained release of diphenhydramine hydrochloride. J. Nanopart. Res. 2015, 17, 218 10.1007/s11051-015-3032-3.

[ref62] Ricci L.; Umiltà E.; Righetti M. C.; Messina T.; Zurlini C.; Montanari A.; Bronco S.; Bertoldo M. On the thermal behavior of protein isolated from different legumes investigated by DSC and TGA. J. Sci. Food Agric. 2018, 98, 5368–5377. 10.1002/jsfa.9078. 29660127

[ref63] Mueller B. Experimental Interactions between Clay Minerals and Bacteria: A Review. Pedosphere 2015, 25, 799–810. 10.1016/s1002-0160(15)30061-8.

[ref64] Alimova A.; Katz A.; Steiner N.; Rudolph E.; Wei H.; Steiner J. C.; Gottlieb P. Bacteria-clay Interaction: Structural Changes in Smectite Induced During Biofilm Formation. Clays Clay Miner. 2009, 57, 205–212. 10.1346/ccmn.2009.0570207.

[ref65] Alimova A.; Coté G. L.; Block K.; Priezzhev A. V.; Rudolph E.; Katz A.; Steiner J. C.; Gottlieb P.; Alfano R. R. Bacteria-clay Interactions Investigated by Light Scattering and Phase Contrast Microscopy. Optical Diagnostics and Sensing VI, 2006; Vol. 6094.

[ref66] Huang Q.; Wu H.; Cai P.; Fein J. B.; Chen W. Atomic Force Microscopy Measurements of Bacterial Adhesion and Biofilm Formation onto Clay-sized Particles. Sci. Rep. 2015, 5, 16857 10.1038/srep16857. 26585552 PMC4653644

[ref67] Zhang Y.; Chen Y.; Zhang H.; Zhang B.; Liu J. Potent Antibacterial Activity of a Novel Silver Nanoparticle-halloysite Nanotube Nanocomposite Powder. J. Inorg. Biochem. 2013, 118, 59–64. 10.1016/j.jinorgbio.2012.07.025. 23123339

[ref68] Abhinayaa R.; Jeevitha G.; Mangalaraj D.; Ponpandian N.; Meena P. Toxic Influence of Pristine and Surfactant Modified Halloysite Nanotubes on Phytopathogenic Bacteria. Appl. Clay Sci. 2019, 174, 57–68. 10.1016/j.clay.2019.03.022.

[ref69] Choi H.-J.; Stazak T. J.; Montemagno C. D. Surface-dependent Cytotoxicity on Bacteria as a Model for Environmental Stress of Halloysite Nanotubes. J. Nanopart. Res. 2013, 15, 2008 10.1007/s11051-013-2008-4.

[ref70] Biswas B.; Warr L. N.; Hilder E. F.; Goswami N.; Rahman M. M.; Churchman J. G.; Vasilev K.; Pan G.; Naidu R. Biocompatible Functionalisation of Nanoclays for Improved Environmental Remediation. Chem. Soc. Rev. 2019, 48, 3740–3770. 10.1039/c8cs01019f. 31206104

