# Supplementary material for: Antibody-Functionalized Halloysite Nanotubes for Targeting Bacterial Cells
Source: ACS Appl Bio Mater. 2021 Apr 11;4(5):4094–104. doi: 10.1021/acsabm.0c01332 (PMC8161669; doi:10.1021/acsabm.0c01332)
Supplement: Supplementary file 1 — mt0c01332_si_001.pdf [file mt0c01332_si_001.pdf]

Supporting Information

Antibody-Functionalized Halloysite Nanotubes for Targeting Bacterial Cells

*Ofer Prinz Setter, Ariel Movsowitz, Sarah Goldberg, and Ester Segal**

Department of Biotechnology and Food Engineering, Technion Israel Institute of Technology, Technion City, 3200003 Haifa, Israel

Correspondence: esegal@technion.ac.il

Table of Contents

Surface modification of HNTs: Background and details	Page S3
Figure S1	Page S4
Figure S2	Page S5
Table S1	Page S5
Figure S3	Page S6
Table S2	Page S7
Figure S4	Page S8
Figure S5	Page S9
Figure S6	Page S10

Surface modification of HNTs: Background and details

To enhance HNTs surface reactivity, acidic etching is performed to enlarge the nanotube specific surface area and expose additional reactive hydroxyl groups[1-3]. Other routes for activation of HNTs reported in the literature include calcination[1, 4-7] and alkali treatment[8-9]; while the former is expected to decrease the number of required hydroxyl groups and the later mostly removes the silica component of the HNTs which is essential for the next synthetic steps. The surface chemical activation of the etched HNTs (E-HNTs) is achieved by their silanization with aminopropyltriethoxysilane (APTES) in toluene based on the highly-cited work by Yuan et al.[10]. Other, less investigated, silanization techniques for HNTs include adding trimethylamine (TEA) to the reaction, as a base catalyst with lower reaction temperature (80 °C)[11], performing the reaction under nitrogen atmosphere[12-13] or the use of different solvents[14]. The next step in the synthetic route is the carboxylation of the surface amine residues through a reaction with succinic anhydride in dimethylformamide (DMF)[13, 15]. The Hydrophilic carboxyl residues could assist in keeping HNTs suspensions more stable without the need of extreme sonication[13]. In addition, carboxyl functionalized surface will readily form amide bond with any amino reagent through the simple and rapid EDC/NHS reaction[16-19] in aqueous medium suitable for most proteins [18]. For protein A conjugation onto the carboxylated surface, a two-step EDC/sulfo-NHS [20-21] reaction in 0.05 M MES buffer pH 6.0 is carried out. The separation into two steps prevents the unwanted protein-protein crosslinking between their own amine and carboxyl residues. The use of the negatively-charged sulfo-NHS ensures good suspension stability for the reactive intermediate. Hydrolysis of the reactive sulfo-NHS ester is minimized by conducting the reaction rapidly in a cold medium at slightly acidic pH [21]. Protein conjugation onto HNTs was also reported using a glutaraldehyde crosslinker in water[22-23]; however, this method suffers from nonuniformity of the glutaraldehyde polymeric species [21, 24] and the intermediate is not well suspended. The conjugation of protein A enables the immobilization of antibodies at a proper orientation significantly enhancing their binding efficacy[25-28].

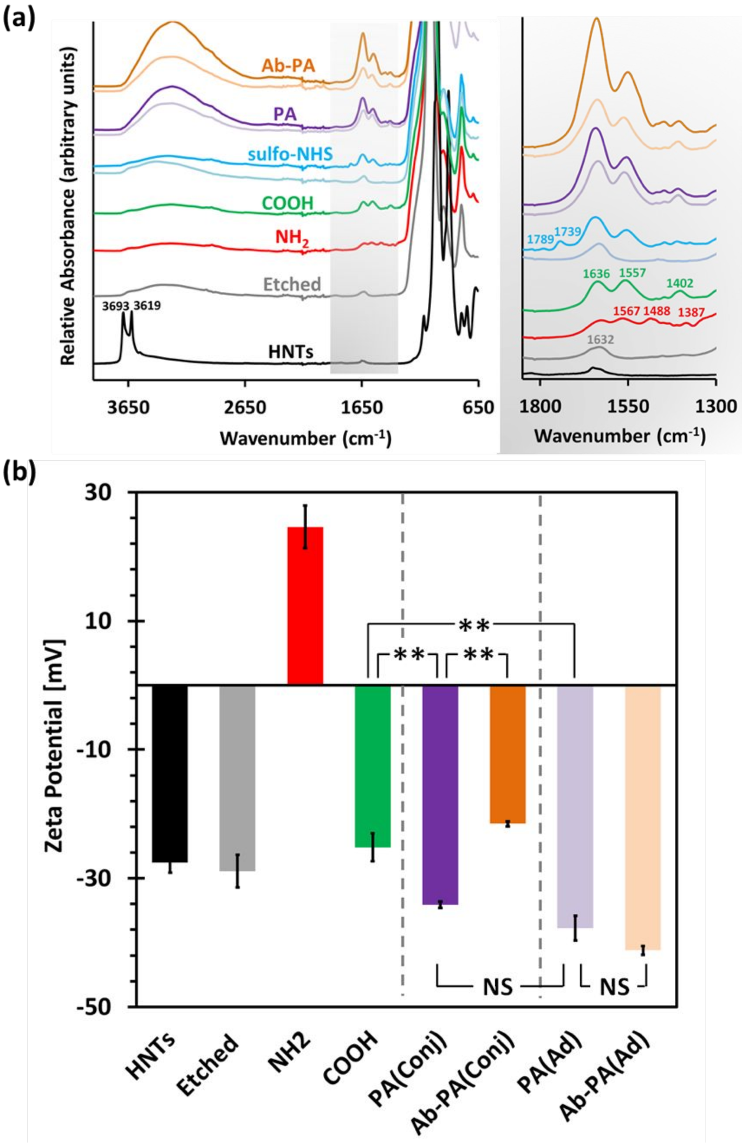

Figure S1. Characterization of the different synthetic steps followed for the oriented immobilization of the antibody onto the HNTs. (a) ATR-FTIR spectra of the HNTs following each of the synthetic steps and the respective controls; all spectra are normalized to inert highest peak. Right panel presents zoom-in spectra at wavenumber range of 1800-1300 cm^{-1}. Black trace for pristine HNTs, grey trace for etched HNTs (E-HNTs), red trace for silanized HNTs (NH_2), green trace for carboxylated HNTs (COOH), blue trace for sulfo-NHS-activated HNTs (sulfo-NHS) and light blue for the respective control when the sulfo-NHS reaction is carried out on etched HNTs (without prior silanization and carboxylation), purple trace for PA-conjugated HNTs (PA) and the light purple is the respective control for adsorbed PA, orange trace for the antibody-immobilized HNTs (Ab-PA) and light orange trace for the control where the antibody was incubated with the PA-adsorbed HNTs. (b) Zeta potential measurements of the HNTs following the different synthesis steps and controls. $^{**}p<0.01$, one-tail t-Test. NS marks $p>0.05$, two-tail t-Test.

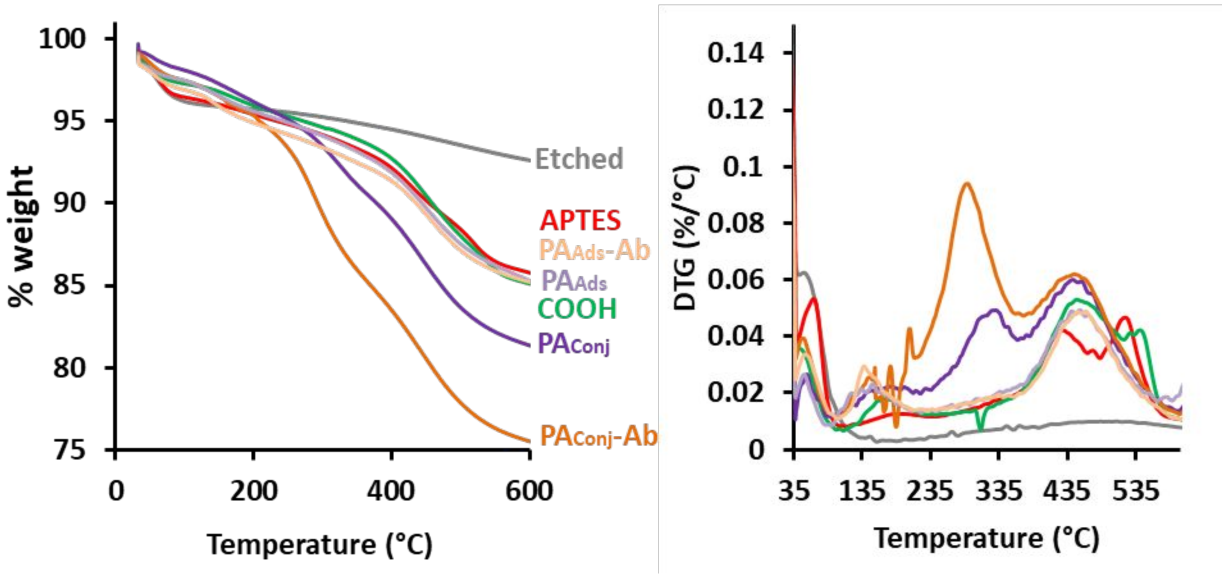

Figure S2. TGA analysis: TGA thermograms (left) and their corresponding derivative curves (right) of – grey trace for etched HNTs (E-HNTs), red trace for silanized HNTs (NH_2), green trace for carboxylated HNTs (COOH), purple trace for PA-conjugated HNTs (PA) and the light purple is the respective control for adsorbed PA, orange trace for the antibody-immobilized HNTs (Ab-PA) and light orange trace for the control where the antibody was incubated with the PA-adsorbed HNTs.

Table S1. Summary of the main weight loss events in TGA analysis.

	Weight loss between 32-110 °C	Weight loss between 110-265 °C	Weight loss between 265-600 °C	Organic increment 110-600 °C *
**Etched HNTs**	3.2±0.2	0.6±0.3	2.8±0.3	-
**APTES**	2.8±0.3	1.7±0.3	8.8±0.5	7.1±0.7
**COOH**	2.3±0.8	2.1±0.3	10±1	1.5±0.9
**PA Conj**	2±2	3±2	13±2	5±3
**PA Conj Ab**	2±2	5±2	17±3	5±4
**PA Ads**	2±1	2.7±0.5	9±1	0±2
**PA Ads Ab**	2.1±0.8	3±0.5	9±1	0±1

*) Organic increment between 110-600 °C was calculated as follows:
$OI_{step\ n} = [WL_{32\text{-}110°C} + WL_{110\text{-}265°C}]_{step\ n} - [WL_{32\text{-}110°C} + WL_{110\text{-}265°C}]_{step\ n\text{-}1}$

Where OI = Organic increment between 110-600 °C; $WL_{32\text{-}110°C}$ = Weight loss between 32-110 °C ; $WL_{110\text{-}265°C}$ = Weight loss between 110-265 °C

For example:
$OI_{APTES} = [WL_{32\text{-}110°C} - WL_{110\text{-}265°C}]_{APTES} - [WL_{32\text{-}110°C} - WL_{110\text{-}265°C}]_{Etched\ HNTs} =$
$(1.7±0.3 + 8.8±0.5) - (0.6±0.3 + 2.8±0.3) = 7.1±0.7$ %wt.

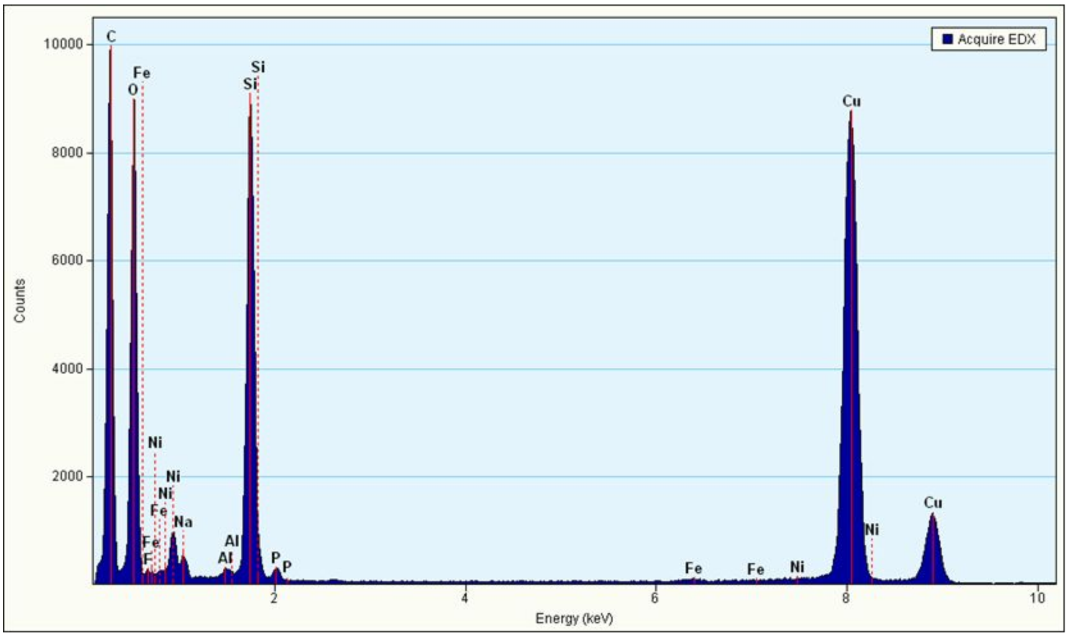

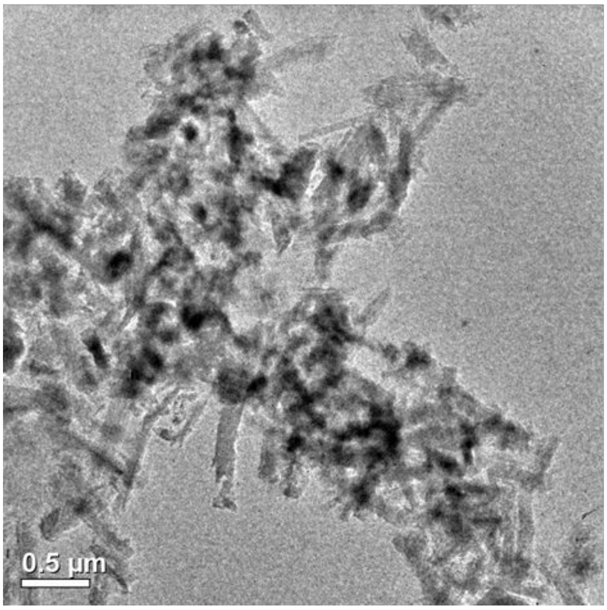

	[Al]	[Si]	[O]
Wt%	0.3	12	16

Al/Si ratio = 0.025

Figure S3. TEM EDX measurement of etched HNTs. EDX spectrum (above) and the TEM image of the measured region (below on the left). Calculated weight percentage for Al, Si and O as well as the Al/Si ratio are displayed in the table below. C and Cu signals are contributed from the TEM grid, whereas Na and P signals are contributed from PBS residues on the etched HNTs. Fe and Ni peak locations are indicated for information only while no significant signal was obtained for these elements.

Table S2. Comparison of Zeta potential values of etched HNTs following their modification as measured in pure water and PBS 0.1 M pH 7.2

	**Etched HNTs**	**Amino-silanized etched HNTs**	**Carboxylated etched HNTs**
In pure water	-29±3	+25±3	-25±2
In 0.1 M PBS pH 7.2	-31±2	-7±1	-35±1

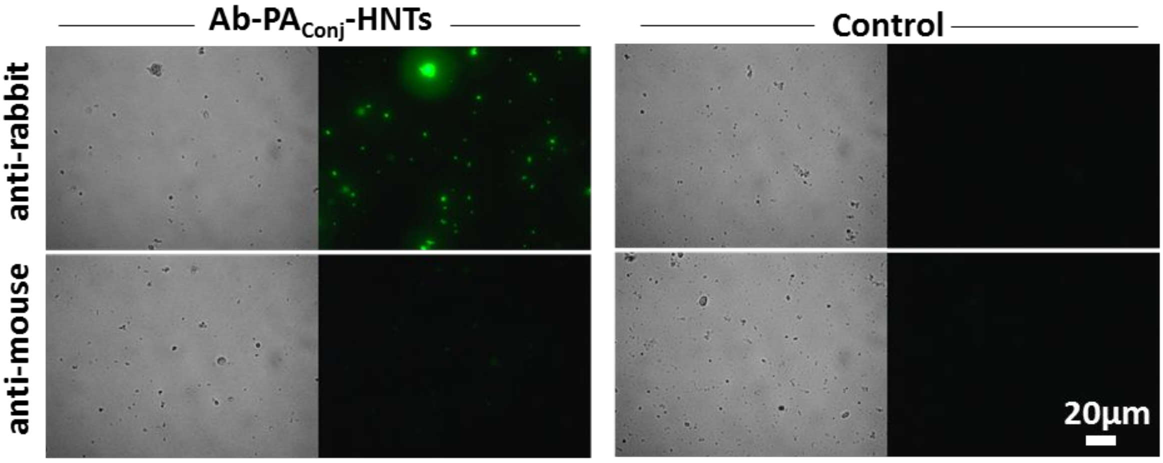

Figure S4. Bright field and corresponding fluorescence micrographs of antibody-functionalized HNTs (left panel) and non-functionalized carboxylated HNTs control (right panel) following immuno-labeling with fluorescein isothiocyanate (FITC)-anti-*rabbit* antibody (upper panel) and FITC-anti-*mouse* antibody (lower panel).

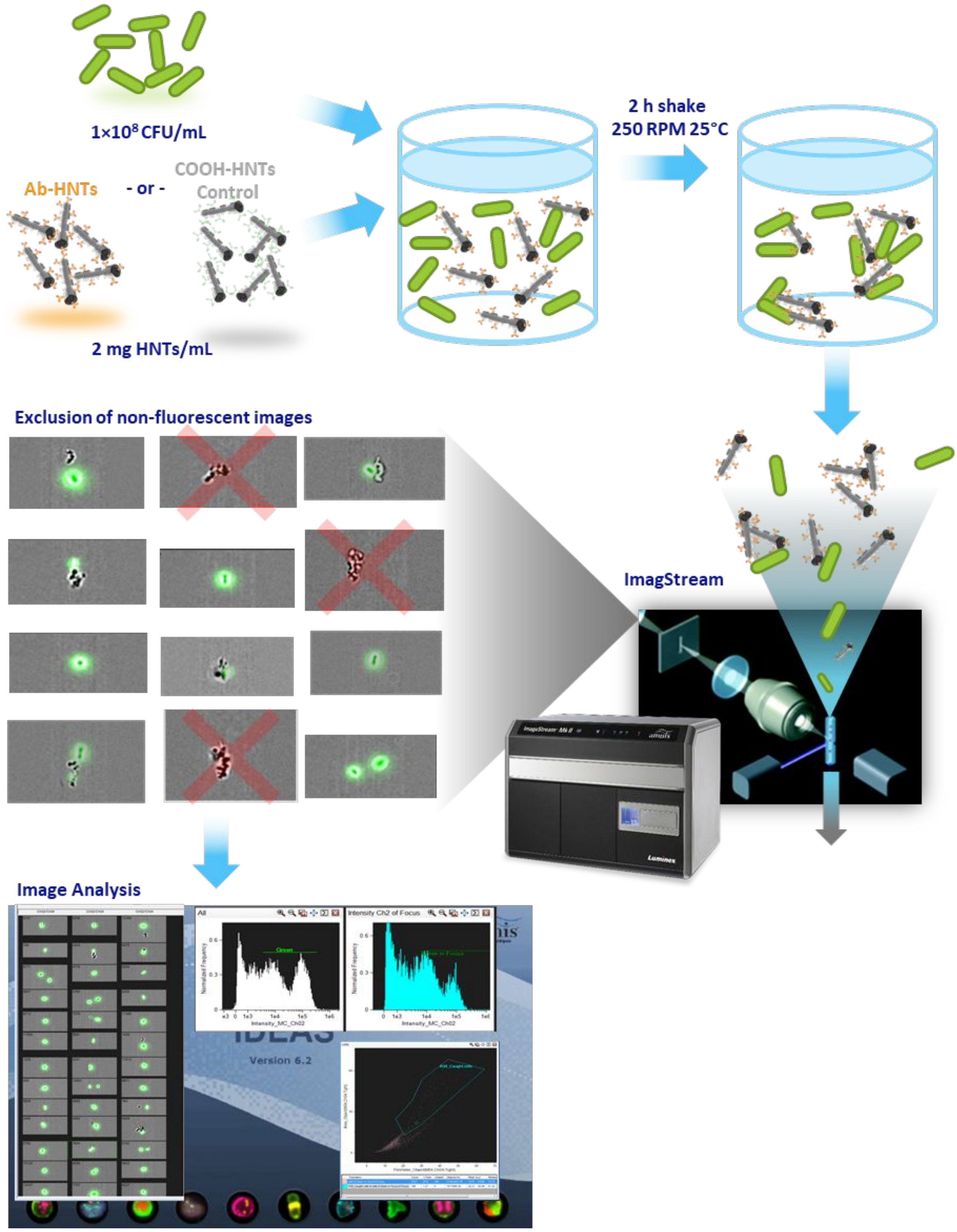

Figure S5. Flow chart for the analytical procedure of high-throughput flow cytometry analysis: The investigated HNTs (antibody-functionalized or no-antibody controls) are mixed with the target bacteria (green fluorescent *E. coli*) and gently shaken for 2 h. Then, the suspension is transferred into the ImageStream instrument where it is passed in a flow cell while each suspended object is imaged separately generating thousands of images. Only images depicting green fluorescent objects in focus are passed forward to image analysis.

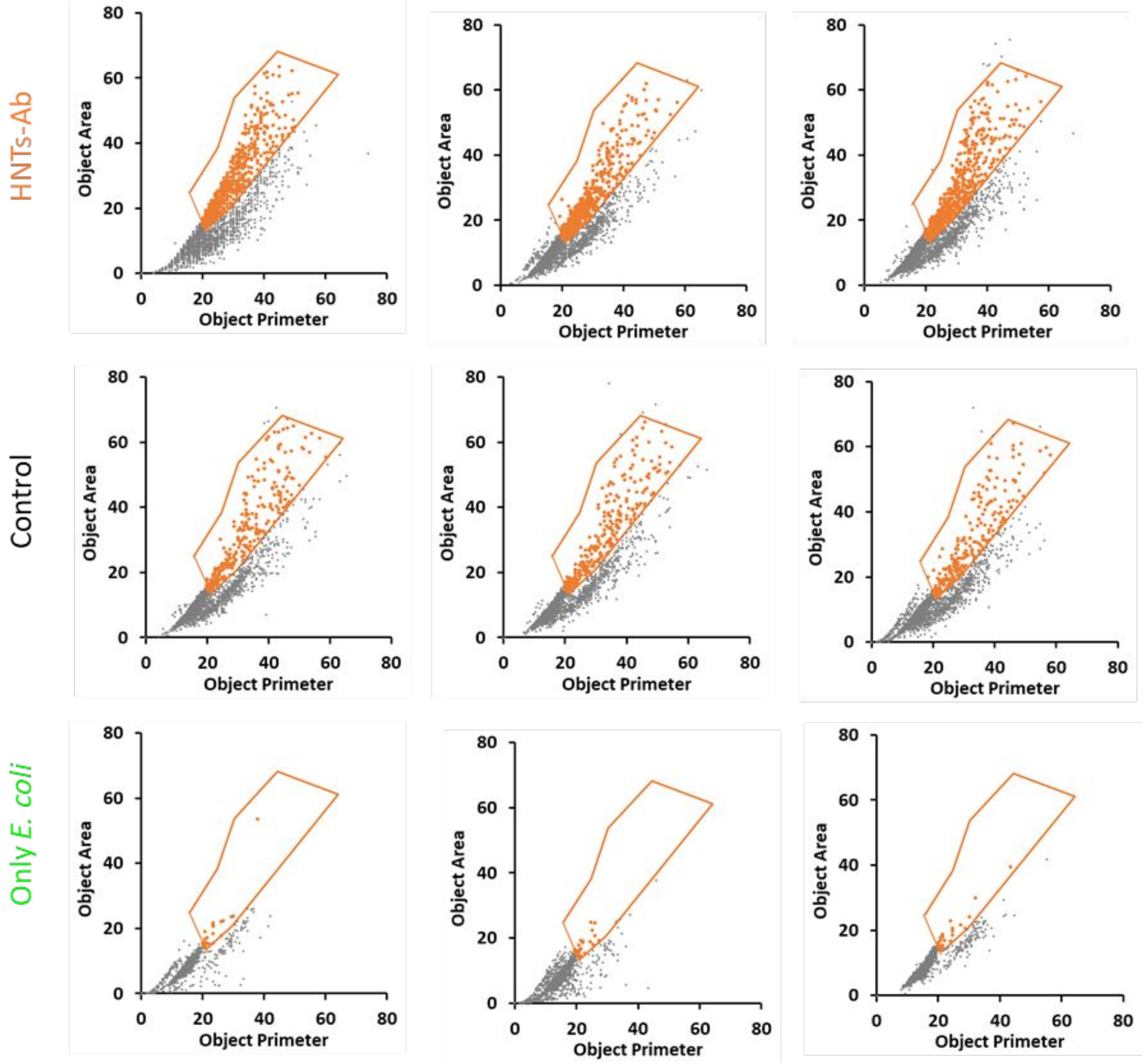

Figure S6. High-throughput flow cytometry measurements. Cytograms of the gated images for all experiments positioned on object area vs. object perimeter plain with a well-defined region of “bound” bacteria (orange gate). All samples were measured after 2 h of gentle shaking at RT in PBS 0.1 M pH 7.2. Upper panel – mixtures of GFP/Amp *E. coli* (K-12, 1x10^8 cell mL^{-1}) and antibody-functionalized HNTs (2 mg mL^{-1}); middle panel – mixtures of GFP/Amp *E. coli* (K-12, 1x10^8 cell mL^{-1}) and no-antibody control carboxylated HNTs (2 mg mL^{-1}); and lower panel – only GFP/Amp *E. coli* (K-12, 1x10^8 cell mL^{-1}).

References

(1) Belkassa, K.; Bessaha, F.; Marouf-Khelifa, K.; Batonneau-Gener, I.; Comparot, J.; Khelifa, A. Physicochemical and Adsorptive Properties of A Heat-treated and Acid-leached Algerian Halloysite. *Colloids Surf. Physicochem. Eng. Aspects* **2013,** *421*, 26-33.
(2) Garcia-Garcia, D.; Ferri, J. M.; Ripoll, L.; Hidalgo, M.; Lopez-Martinez, J.; Balart, R. Characterization of Selectively Etched Halloysite Nanotubes by Acid Treatment. *Appl. Surf. Sci.* **2017,** *422*, 616-625.
(3) Sun, P.; Liu, G.; Lv, D.; Dong, X.; Wu, J.; Wang, D. Effective Activation of Halloysite Nanotubes by Piranha Solution for Amine Modification via Silane Coupling Chemistry. *RSC Adv.* **2015,** *5* (65), 52916-52925.
(4) Aytekin, M. T.; Hoşgün, H. L. Characterization studies of heat-treated halloysite nanotubes. *Chemical Papers* **2020**.
(5) Deng, L.; Yuan, P.; Liu, D.; Du, P.; Zhou, J.; Wei, Y.; Song, Y.; Liu, Y. Effects of calcination and acid treatment on improving benzene adsorption performance of halloysite. *Appl. Clay Sci.* **2019,** *181*.
(6) Jia, S.; Fan, M. Silanization of heat-treated halloysite nanotubes using γ-aminopropyltriethoxysilane. *Appl. Clay Sci.* **2019,** *180*.
(7) Yuan, P.; Tan, D.; Aannabi-Bergaya, F.; Yan, W.; Fan, M.; Liu, D.; He, H. Changes in Structure, Morphology, Porosity, and Surface Activity Of Mesoporous Halloysite Nanotubes Under Heating. *Clays Clay Miner.* **2012,** *60* (6), 561-573.
(8) Khalifa, A. Z.; Cizer, Ö.; Pontikes, Y.; Heath, A.; Patureau, P.; Bernal, S. A.; Marsh, A. T. M. Advances in alkali-activation of clay minerals. *Cem. Concr. Res.* **2020,** *132*, 106050.
(9) Wang, Q.; Zhang, J.; Wang, A. Alkali activation of halloysite for adsorption and release of ofloxacin. *Appl. Surf. Sci.* **2013,** *287*, 54-61.
(10) Yuan, P.; Southon, P. D.; Liu, Z.; Green, M. E. R.; Hook, J. M.; Antill, S. J.; Kepert, C. J. Functionalization of Halloysite Clay Nanotubes by Grafting with γ-aminopropyltriethoxysilane. *J. Phys. Chem. C* **2008,** *112* (40), 15742-15751.
(11) Wu, Y.; Yang, J.; Gao, H.; Shen, Y.; Jiang, L.; Zhou, C.; Li, Y.; He, R.-R.; Liu, M. Folate-conjugated Halloysite Nanotubes, an Efficient Drug Carrier, Deliver Doxorubicin for Targeted Therapy of Breast Cancer. *ACS Appl. Nano Mater.* **2018,** *1* (2), 595-608.
(12) Zeraatpishe, L.; Mohebali, A.; Abdouss, M. Fabrication and characterization of biocompatible pH responsive halloysite nanotubes grafted with sodium alginate for sustained release of phenytoin sodium. *New J. Chem.* **2019,** *43* (26), 10523-10530.
(13) Joo, Y.; Jeon, Y.; Lee, S. U.; Sim, J. H.; Ryu, J.; Lee, S.; Lee, H.; Sohn, D. Aggregation and Stabilization of Sarboxylic Acid Functionalized Halloysite Nanotubes (HNT-COOH). *J. Phys. Chem. C* **2012,** *116* (34), 18230-18235.
(14) Osipova, V. A.; Pestov, A. V.; Mekhaev, A. V.; Abuelsoad, A. M. A.; Tambasova, D. P.; Antonov, D. O.; Kovaleva, E. G. Functionalization of Halloysite by 3-Aminopropyltriethoxysilane in Various Solvents. *Pet. Chem.* **2020,** *60* (5), 597-600.
(15) Liu, M.; Chang, Y.; Yang, J.; You, Y.; He, R.; Chen, T.; Zhou, C. Functionalized Halloysite Nanotube by Chitosan Grafting for Drug Delivery of Curcumin to Achieve Enhanced Anticancer Efficacy. *J. Mater. Chem. B* **2016,** *4* (13), 2253-2263.
(16) He, R.; Liu, M.; Shen, Y.; Long, Z.; Zhou, C. Large-area Assembly of Halloysite Nanotubes for Enhancing the Capture of Tumor Cells. *J. Mater. Chem. B* **2017,** *5* (9), 1712-1723.
(17) He, R.; Liu, M.; Shen, Y.; Liang, R.; Liu, W.; Zhou, C. Simple Fabrication of Rough Halloysite Nanotubes Coatings by Thermal Spraying for High Performance Tumor Cells Capture. *Mater. Sci. Eng., C* **2018,** *85*, 170-181.

(18) Zhao, Q.; Liu, C.; Liu, J.; Zhang, Y. Development of a Novel Polyethersulfone Ultrafiltration Membrane with Antibacterial Activity and High Flux Containing Halloysite Nanotubes Loaded with Lysozyme. *RSC Adv.* **2015,** *5* (48), 38646-38653.
(19) Bonfield, T. L.; John, N.; Barna, B. P.; Kavuru, M. S.; Thomassen, M. J.; Yen-Lieberman, B. Multiplexed Particle-based Anti-granulocyte Macrophage Colony Stimulating Factor Assay Used as Pulmonary Diagnostic test. *Clin. Diagn. Lab. Immunol.* **2005,** *12* (7), 821-4.
(20) Yamina, A. M.; Fizir, M.; Itatahine, A.; He, H.; Dramou, P. Preparation of Multifunctional PEG-graft-halloysite Nanotubes for Controlled Drug Release, Tumor Cell Targeting, and Bio-imaging. *Colloids Surf. B. Biointerfaces* **2018,** *170*, 322-329.
(21) Hermanson, G. T. Chapter 14 - Microparticles and Nanoparticles. In *Bioconjugate Techniques (Third Edition)*; Hermanson, G. T., Ed.; Academic Press: Boston, 2013; pp 549-587.
(22) Kadam, A. A.; Jang, J.; Lee, D. S. Supermagnetically Tuned Halloysite Nanotubes Functionalized with Aminosilane for Covalent Laccase Immobilization. *ACS Appl Mater Interfaces* **2017,** *9* (18), 15492-15501.
(23) Kim, M.; Jee, S. C.; Sung, J. S.; Kadam, A. A. Anti-proliferative applications of laccase immobilized on super-magnetic chitosan-functionalized halloysite nanotubes. *Int. J. Biol. Macromol.* **2018,** *118* (Pt A), 228-237.
(24) Hermanson, G. T. Chapter 5 - Homobifunctional Crosslinkers. In *Bioconjugate Techniques (Third Edition)*; Hermanson, G. T., Ed.; Academic Press: Boston, 2013; pp 275-298.
(25) Hermanson, G. T. Chapter 15 - Immobilization of Ligands on Chromatography Supports. In *Bioconjugate Techniques (Third Edition)*; Hermanson, G. T., Ed.; Academic Press: Boston, 2013; pp 589-740.
(26) Demirel, G.; Çaykara, T.; Akaoğlu, B.; Çakmak, M. Construction of a novel multilayer system and its use for oriented immobilization of immunoglobulin G. *Surf. Sci.* **2007,** *601* (19), 4563-4570.
(27) Boyle, M. D. P.; Reis, K. J. Bacterial Fc Receptors. *Nat. Biotechnol.* **1987,** *5* (7), 697-703.
(28) Arshavsky-Graham, S.; Urmann, K.; Salama, R.; Massad-Ivanir, N.; Walter, J. G.; Scheper, T.; Segal, E. Aptamers vs. antibodies as capture probes in optical porous silicon biosensors. *Analyst* **2020**, 4991-5003.
